# Ionic Liquid‐Based Electrolyte with Multiple Hydrogen Bonding Network Enabling High‐Voltage Stable Proton Batteries Across Wide Temperature Range

**DOI:** 10.1002/advs.202416931

**Published:** 2025-02-14

**Authors:** Xiaoyu Dong, Zhiwei Li, Hai Xu, Zhiyuan Wu, Fanhao Meng, Shuzhi Liu, Hui Dou, Xiaogang Zhang

**Affiliations:** ^1^ Jiangsu Key Laboratory of Materials and Technologies for Energy Storage Technology College of Material Science and Technology Nanjing University of Aeronautics and Astronautics Nanjing 210016 P. R. China; ^2^ Chemical and Biomolecular Engineering National University of Singapore Singapore 117585 Singapore

**Keywords:** high‐voltage, ionic liquid electrolyte, multi‐level hydrogen bonding network, proton batteries

## Abstract

Proton batteries are strong contender for next‐generation energy storage due to their high safety and rapid response. However, the narrow electrochemical window of acidic aqueous electrolytes limits their energy density and stability. Here, an ionic liquid (IL)‐based electrolyte (EMImOTf‐H_3_PO_4_) containing H_3_PO_4_ in polar IL solvent 1‐ethyl‐3‐methylimidazolium trifluoromethanesulfonate (EMImOTf) is developed for stable high‐voltage energy storage. H_3_PO_4_ serving as a proton source interacts with both EMIm^+^ and OTf^−^, forming an intricate hydrogen bonding network that effectively prevents electrolyte decomposition at high voltage. The half‐cell in EMImOTf‐H_3_PO_4_ electrolyte and pre‐protonated vanadium hexacyanoferrate (H‐VHCF) cathode demonstrates a 126% improvement in Coulombic efficiency over aqueous electrolytes at a current density of 1 A g^−1^. The fabricated PTCDA/MXene//EMImOTf‐H_3_PO_4_//H‐VHCF full battery achieves an operating voltage of 2 V at room temperature, surpassing currently reported values for proton batteries. After 30 000 cycles at 5 A g^−1^, the battery retains 86.1% of its initial capacity. It delivers an energy density of 87.5 Wh kg^−1^ and a power density of 30.6 kW kg^−1^ at room temperature, and can maintain stable operation across a temperature range of 110 °C (−60 ∼ 50 °C). These findings present new possibilities for proton batteries in all‐weather grid‐scale energy storage applications.

## Graphical abstract

An ionic liquid electrolyte with multilane hydrogen‐bond interactions is designed for stable, high‐voltage proton storage. Such enhanced hydrogen bond imparts rapid and stable electrochemical proton storage with a high operating voltage of 2 V. A symmetric full proton battery using the electrolyte achieves a maximum energy density of 87.5 Wh kg^−1^ and a maximum power density of 30.6 kW kg^−1^.

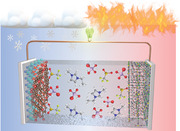

## 1 Introduction

Given the exceptionally abundant hydrogen reserves on Earth, proton batteries are considered an optimal choice for large‐scale and low‐cost energy storage. The rate and capacity performance of energy storage devices are closely related to the transport and storage of charge carries within the electrolyte and electrode. Therefore, the selection of charge carries is crucial. Considerable progress has been made in batteries that use metal ion as charge carries, including lithium‐ion, sodium‐ion, potassium‐ion, zinc‐ion batteries.^[^ ^1^, ^2^ ^]^ However, the transport rate of metal ions is limited by their size. In contrast, proton (H^+^), with the lowest atomic mass and smallest ionic radius, can migrate quickly within the electrolyte. Protons serve as a viable alternative to improve battery performance, although they have received less attention and require further investigation to improve batteries performance.^[^ ^3^, ^4^, ^5^ ^]^ During the insertion and extraction processes, the small size of H^+^ enables rapid reaction kinetic while minimizing structure strain, thereby extending the cycling life of proton battery. Using lightweight H^+^ instead of metal ion as charge carries can achieve high energy density by reducing the overall weight of battery system.

Up to now, insight into proton batteries has largely focused on research into acidic aqueous solutions. However, the low decomposition potential of water (≈1.23 V) significantly limits the operating voltage of proton batteries to below 1.5 V, thereby constraining the energy density of these batteries.^[^ ^6^ ^]^ In addition, bubbles generated during water decomposition adhere to the electrode surface, reducing electrode stability by obstructing the contact between the electrolyte and the electrode or by reacting with the electrode. Several strategies have been explored to address these challenges. One route involves incorporating molecular crowding into acidic aqueous solution.^[^ ^7^ ^]^ These molecular crowding additives reduce water activity by competing with water molecules for hydrogen bonds, thereby extending the operating voltage of the electrolyte. The other notable approach is the use of “water‐in‐sugar” electrolytes, which form a thin organic film on the electrode surface.^[^ ^8^ ^]^ This film acts as a protective barrier, preventing free water in the electrolyte directly from contacting the electrode and thereby mitigating the corrosive effects of hydrated ions on the electrode material. However, the presence of a large amount of active water in aqueous electrolytes still leads to significant detrimental side reactions, which drastically reduce battery capacity. In contrast, non‐aqueous organic electrolytes offer a promising alternative to overcome the above issues by minimizing or eliminating the presence of active water. But the compatibility of inorganic acids with organic solvents become a huge challenge to develop suitable electrolyte for stable proton storage. Ionic liquids (IL) exhibit excellent acid solubility and chemical stability, making them effective alternatives to organic solvents. Consequently, using IL to construct electrolytes presents significant potential. Nevertheless, the high viscosity and complex ionic structure of IL result in the poor compatibility with electrode materials and low ionic conductivity. Thus, it is vital to select IL that not only mix effectively with acids but are also compatible with electrode materials.

In this work, we present a simple yet powerful strategy for developing electrolytes based on non‐aqueous ILs solvent, aimed at achieving the stable proton storage under high operating voltages and across a wide temperature range. The ILs electrolyte is composed of 1‐ethyl‐3‐methylimidazolium trifluoromethanesulfonate (EMImOTf) as the solvent and phosphoric (H_3_PO_4_) serving as the proton source. On one hand, the interaction between charged ions (EMIm⁺ and OTf^−^) and H_3_PO_4_ creates a complex multilane hydrogen bonding network, which enhances the stability of electrolyte. The local electric fields generated by EMIm^+^ and OTf^−^ guide proton migration along the direction of the electric field, optimizing the proton transport pathway and ensuring efficient proton transfer. On the other hand, EMImOTf effectually alleviates the electrolyte decomposition by reducing the active water, thereby extending the electrochemical stability window (ESW) for proton storage. Molecular dynamics (MD) simulations reveal the presence of complex hydrogen bonding network (i.e., O─H…N and O─H…O) and multiple interactions within the EMImOTf‐H_3_PO_4_ system. The proton battery in EMImOTf‐H_3_PO_4_ electrolyte and pre‐protonated vanadium hexacyanoferrate (H‐VHCF) cathode achieves a Coulombic efficiency (CE) of 92.6% at 1 A g^−1^, which is a 126% improvement over the traditional H_2_O‐H_3_PO_4_ aqueous electrolyte. This system shows stable cycling for 10 000 cycles at 5 A g^−1^, ten times the 1000 cycles of H_2_O‐H_3_PO_4_ system. The PTCDA/MXene//EMImOTf‐H_3_PO_4_//H‐VHCF full proton battery can operate at a high voltage of 2 V at room temperature and retains 86.1% of its initial capacity after 30 000 cycles. Furthermore, the EMImOTf‐H_3_PO_4_ electrolyte demonstrates excellent stability under extreme temperatures, allowing the battery to function reliably from 50 to −60 °C. This performance highlights its potential for use in harsh environments and provides vital evidence for future energy storage solutions in various climatic conditions.

## 2 Results and Discussion

### 2.1 Characterization of Electrolytes

**Figure 1:**
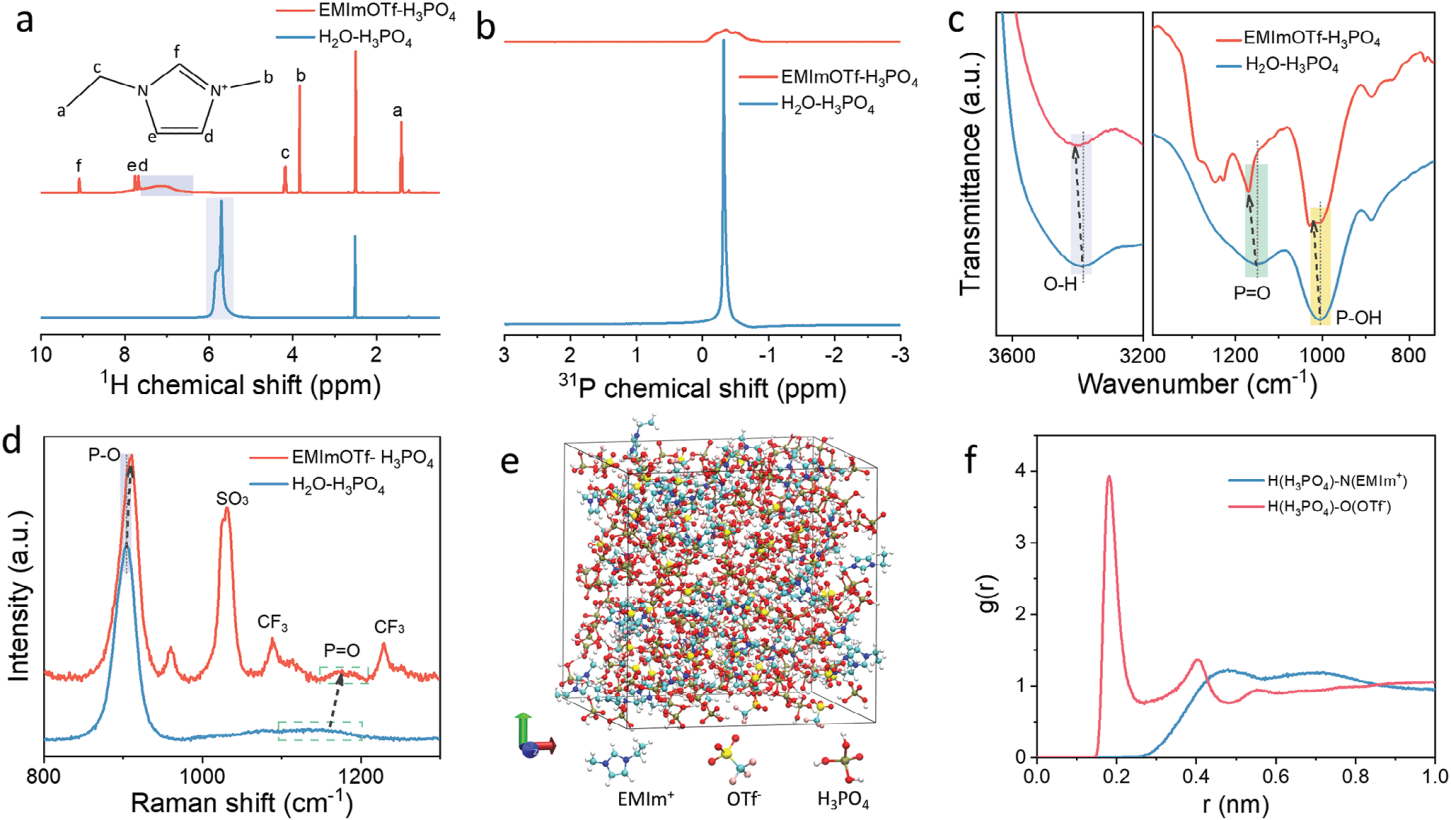
Characterization of EMImOTf‐H_3_PO_4_ and H_2_O‐H_3_PO_4_. a) ^1^H NMR, b) ^31^P NMR, c) FT‐IR, and d) Raman spectra. e) The snapshot of EMImOTf‐H_3_PO_4_ electrolyte from MD simulation. f) The RDFs for H (H_3_PO_4_)‐N (EMIm^+^) and H (H_3_PO_4_)‐O (OTf^−^).

Traditional aqueous acidic electrolytes often lead to side reactions, which significant challenge the stability of proton batteries. An IL‐based nonaqueous proton‐conducting electrolyte containing H_3_PO_4_ in polar ionic liquid solvent EMImOTf was rationally designed to overcome this issue. The interplay between H_3_PO_4_ and EMImOTf was investigated by a series of spectroscopic methods. The ^1^H nuclear magnetic resonance (^1^H NMR) spectra of EMImOTf‐H_3_PO_4_ and H_2_O‐H_3_PO_4_ are shown in **Figure** **1**a. A narrow and sharp signal at 5.7 ppm in the ^1^H NMR spectrum of H_2_O‐H_3_PO_4_ is assigned to hydrogen atom of H_3_PO_4_, which becomes broad and weak in EMImOTf‐H_3_PO_4_, indicating interaction diversity between H_3_PO_4_ and EMImOTf.^[^ ^9^, ^10^ ^]^ Additionally, the ^1^H signal of H_3_PO_4_ shifts downfield in EMImOTf‐H_3_PO_4_ due to the enhanced hydrogen‐bond donor capability of H_3_PO_4_, which decreases the electron density around the hydrogen atoms and thereby weakens the shielding effect.^[^ ^11^, ^12^, ^13^ ^]^ Figure 1b shows a broader and weaker ^31^P signal for EMImOTf‐H_3_PO_4_ compared to H_2_O‐H_3_PO_4_, underscoring the intricate and varied chemical environment surrounding H_3_PO_4_.^[^ ^14^ ^]^ The O─H vibration of H_3_PO_4_ occurs at 3400 cm^−1^ in the Fourier transform infrared spectroscopy (FT‐IR) spectrum of H_2_O‐H_3_PO_4_. Interestingly, this peak shifts to 3410 cm^−1^ in the FT‐IR spectrum of EMImOTf‐H_3_PO_4_, indicating the strengthening of the covalent bonds in phosphoric acid. Moreover, the FT‐IR peaks corresponding to P═O and P─OH vibrations observed at 1150 and 998 cm^−1^ for H_2_O‐H_3_PO_4_ shift to 1170 and 1025 cm^−1^ for EMImOTf‐H_3_PO_4_ (Figure 1c; Figure S1, Supporting Information). This suggests the enhanced strength of the P═O and P─O─H bonds in EMImOTf‐H_3_PO_4_, which contribute to the improved structural stability of the electrolyte.^[^ ^15^ ^]^ Assignment of other significant FT‐IR peaks of EMImOTf‐H_3_PO_4_ are show in Figure S1 and Table S1 (Supporting Information).^[^ ^16^ ^]^ In the Raman spectrum of H_2_O‐H_3_PO_4_, the peak at 904 cm^−1^ is primarily due to the symmetric stretching of P─O bonds in H_3_PO_4_, with a minor contribution from H_2_PO_4_^−^, while the peak at 1132 cm^−1^ corresponds to the stretching of P═O bond. In EMImOTf‐H_3_PO_4_, these peaks shift to 910 and 1177 cm^−1^, respectively, revealing an enhancement in the stability of H_3_PO_4_ within the electrolyte (Figure 1d; Figure S2, Supporting Information).^[^ ^17^, ^18^ ^]^ Additionally, the Raman peaks at 1030, 1088, and 1288 cm^−1^ of EMImOTf‐H_3_PO_4_ correspond to the symmetric stretching vibrations of ‐SO_3_^−^, antisymmetric stretching vibration of ‐CF_3_, and symmetric stretching vibrations of ‐SO_3_^−^, respectively.^[^ ^19^, ^20^ ^]^ Snapshots of the electrolyte systems at equilibrium after 20 ns are shown in Figures 1e and S3 (Supporting Information). Figure S4a (Supporting Information) presents the mean square displacement (MSD) curves for H_3_PO_4_, EMIm^+^, and OTf^−^, with diffusion coefficients of 0.0820×10^−5^, 0.0378×10^−5^, and 0.0624×10^−5^ cm^2^ s^−1^, respectively. These results indicate that EMIm^+^ and OTf^−^ create a stable solvent environment for the proton conduction. The radial distribution function (RDFs) for H (H_3_PO_4_)‐O (OTf^−^) exhibit three distinct peaks at 0.2, 0.4, and 0.6 nm, indicting three different levels of hydrogen–oxygen interactions. This reflects the diversity and order of hydrogen bonding, enhancing the stability and efficiency of proton migration. The RDFs for H (H_3_PO_4_)‐N (EMIm^+^) display two peaks at 0.5 and 0.7 nm, suggesting weakened hydrogen–nitrogen interaction, which still contributes to the overall order of the hydrogen bond (Figure 1f). These results indicate that the hydrogen bond network in EMImOTf‐H_3_PO_4_ features the diversity and order, which could enhance the electrolyte stability as well as maintain the efficient proton transport.^[^ ^21^ ^]^ Further analysis reveals the hydrogen bonds between H_3_PO_4_ and OTf^−^ play a predominant role although the presence of hydrogen bond between H_3_PO_4_ and EMIm^+^ (Figure S4b, Supporting Information). Furthermore, H_3_PO_4_ exhibits a more concentrated distribution around H_2_O, while H_2_O are more dispersed around H_3_PO_4_, with weaker interactions (Figure S5, Supporting Information). The intricate hydrogen bond network significantly enhances the overall stability of EMImOTf‐H_3_PO_4_ electrolyte and optimizes the proton migration pathways, leading to improved performance in practical applications.

### 2.2 Electrochemical Performance of EMImOTf‐H_3_PO_4_

**Figure 2:**
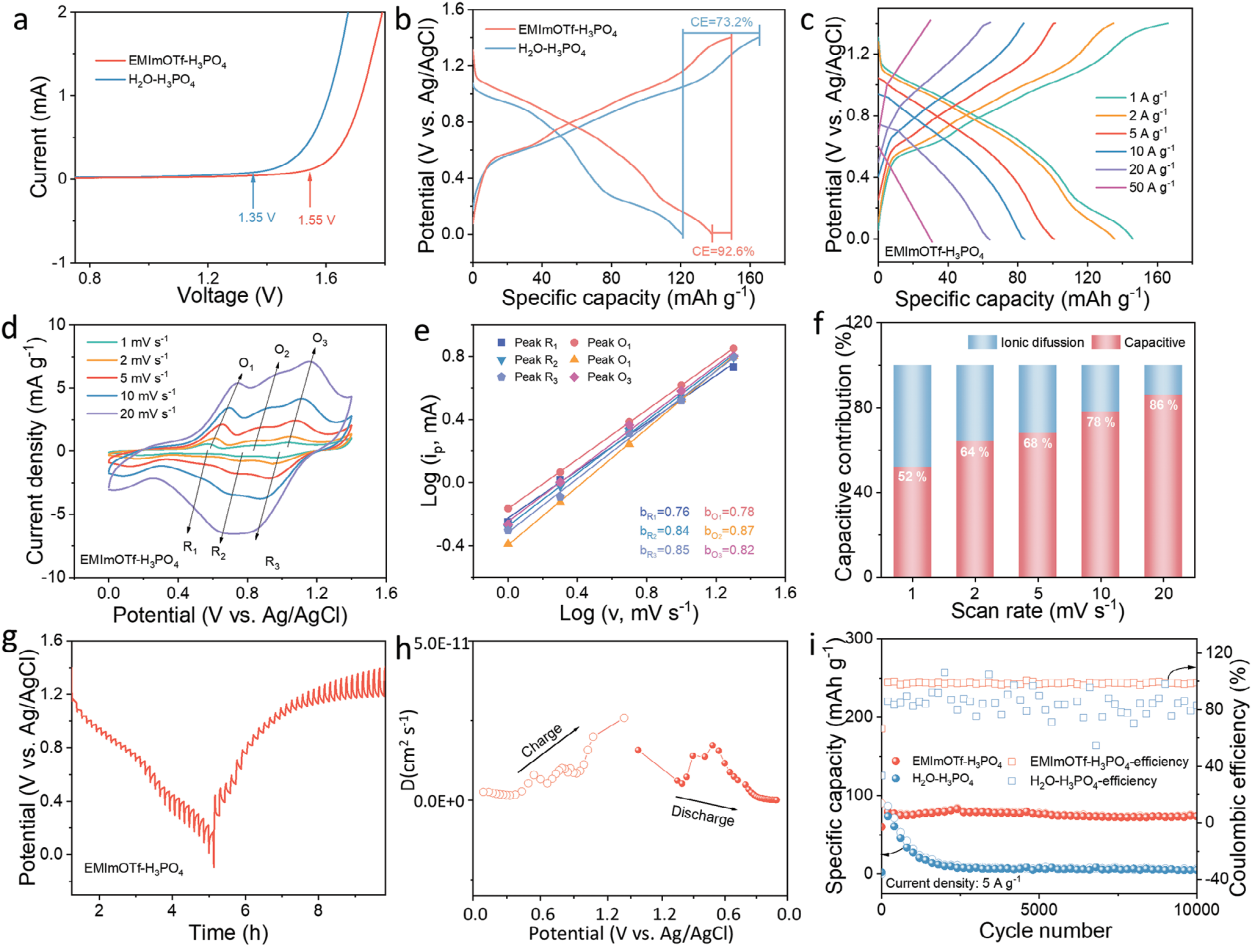
Electrochemical performance of EMImOTf‐H_3_PO_4_ evaluated with a H‐VHCF cathode in half‐cell. a) LSV profiles of different electrolytes in a stainless‐steel symmetrical battery. GCD curves in b) different electrolytes at 1 A g^−1^ and c) EMImOTf ‐H_3_PO_4_ at different current densities. d) CV curves at various scan rates. e) Logarithmic plots of current (*i*) versus scan rate (*v*). f) The ratios of capacitance contribution at different scan rates. g) GITT curves and h) corresponding $D_{H^{+}}$. i) Long‐term cycling stability of H‐VHCF in different electrolytes.

The ESWs of EMImOTf‐H_3_PO_4_ and H_2_O‐H_3_PO_4_ were measured via linear sweep voltammetry (LSV) using a stainless‐steel symmetrical proton battery. As shown in **Figure** **2**a, the decomposition voltage of the EMImOTf‐H_3_PO_4_ electrolyte is 1.55 V, which is extended by 0.2 V compared to H_2_O‐H_3_PO_4_ electrolyte. The three‐electrode test results show that the EMImOTf‐H_3_PO_4_ electrolyte remains stable until 2.5 V on the anodic side without any signs of decomposition, which is higher than the 1.9 V of H_2_O‐H_3_PO_4_. On the cathodic side, the decomposition potential of EMImOTf‐H_3_PO_4_ is −0.6 V, slightly lower than −0.5 V of H_2_O‐H_3_PO_4_. Additionally, the polarization current of EMImOTf‐H_3_PO_4_ is significantly lower than that of H_2_O‐H_3_PO_4_, indicating stronger suppression to the hydrogen evolution reaction (Figure S6, Supporting Information). SEM tests on stainless steel and titanium show that EMImOTf‐H_3_PO_4_ effectively inhibit corrosion compared to aqueous systems (Figures S7 and S8, Supporting Information). A H‐VHCF cathode was applied for the half‐cell to evaluate the utility of electrolyte. Detailed characterization of H‐VHCF is presented in Figure S9 (Supporting Information). The cyclic voltammetry (CV) curves at 5 mV s^−1^ show that the oxidation and reduction peaks of H‐VHCF in EMImOTf‐H_3_PO_4_ shift to higher and lower potential respectively in comparison to those in H_2_O‐H_3_PO_4_, indicating that oxidation occurs at a higher potential, while reduction takes place at a lower potential in this system (Figure S10, Supporting Information). This change broadens the battery's voltage window, which helps increase the energy density and improve the stability. Galvanostatic charge–discharge (GCD) tests at a current density of 1 A g^−1^ were conducted respectively with EMImOTf‐H_3_PO_4_ and H_2_O‐H_3_PO_4_ electrolytes (Figure 2b). In an EMImOTf‐H_3_PO_4_ electrolyte, the H‐VHCF achieves an elevated CE of 92.6%, representing a 126% improvement over in a H_2_O‐H_3_PO_4_ electrolyte (73.2%). As the current density increases, the CE of H‐VHCF with EMImOTf‐H_3_PO_4_ approaches 100% (Figure S11, Supporting Information). This enhancement is attributed to the alleviation of side reactions at high potential resulting from the improved stability of EMImOTf‐H_3_PO_4_ electrolyte. The H‐VHCF in EMImOTf‐H_3_PO_4_ displays the discharge capacities of 145.9, 135.5, 100.9, 84.2, 63.9, and 30.8 mAh g^−1^ at 1, 2, 5, 10, 20, and 50 A g^−1^, respectively (Figure 2c).While H‐VHCF in H_2_O‐H_3_PO_4_ delivers the specific capacities of 124.5, 119.9, 106.5, 89.1, 71.3, 41.7 mAh g^−1^ at 1, 2, 5, 10, 20, and 50 A g^−1^, respectively (Figure S12, Supporting Information). Despite the proton conductivity of EMImOTf‐H_3_PO_4_ is only 28.3 mS cm^−1^, much lower than 206.5 mS cm^−1^ of H_2_O‐H_3_PO_4_, the rate performance of battery shows only a modest difference, owing to the stable hydrogen bond network and low interfacial activity of EMImOTf‐H_3_PO_4_, which effectively suppresses the side reactions and maintains the efficient ion transport (Figure S13, Supporting Information). Notably, after cycling at high current densities and subsequently returning to 1 A g^−1^, the capacity retention of H‐VHCF in EMImOTf‐H_3_PO_4_ is 94.4%, which is higher than 79.9% in H_2_O‐H_3_PO_4_ electrolyte (Figure S14, Supporting Information). This difference highlights the superior stability of EMImOTf‐H_3_PO_4_ electrolyte during the rate charge–discharge processes. CV curves at scan rates from 1 to 20 mV s^−1^ are shown in Figure 2d. Three distinct pairs of redox peaks at 0.64/0.54 V, 0.87/0.73 V, and 1.07/0.95 V (vs Ag/AgCl) correspond to the V^3+^↔V^4+^+e^−^, Fe^2+^↔Fe^3+^+e^−^, and V^4+^↔V^5+^+e^−^ redox reactions, respectively.^[^ ^22^, ^23^, ^24^ ^]^ Theoretical analysis of the peak current (*i*) response to scan rate (*v*) follows the equation *i*  = *av^b^* , where *b* = 0.5 indicates an ionic diffusion‐controlled process and *b* = 1 signifies a capacitance‐dominated process.^[^ ^25^, ^26^ ^]^ As illustrated in Figure 2e, the *b* values of O_1_/R_1_, O_2_/R_2_, and O_3_/R_3_ are 0.76/0.78, 0.84/0.87, and 0.85/0.82, respectively, suggesting that rapid proton storage via the Grotthuss mechanism exhibits capacitive‐like behavior. With the increase of scan rate, the capacitive contribution rises due to the rapid electrochemical process, while diffusion‐controlled Faradaic reactions are limited at high scan rates. At the scan rate of 20 mV s^−1^, the capacitance contribution reaches 86% (Figure 2f). Galvanostatic intermittent titration technique (GITT) was applied to investigate the diffusion coefficients of H^+^ ($D_{H^{+}}$) during the proton insertion/extraction process (Figure 2g, Figure S15, Supporting Information).^[^ ^27^ ^]^ The calculated range for $D_{H^{+}}$ is from 10^−11^ to 10^−12^ (Figure 2h). Although the proton diffusion coefficient in EMImOTf‐H_3_PO_4_ is somewhat lower compared to traditional aqueous electrolyte, the efficient proton conduction pathways and minimized side reactions mitigate this limitation. Consequently, the battery exhibits excellent electrochemical performance and stability. Long‐term cycle tests at 5 A g^−1^ demonstrate that the batteries with EMImOTf‐H_3_PO_4_ electrolyte retain a specific capacity of 74.4 mAh g^−1^ with a capacity retention of 94% after 10 000 cycles. In contrast, the batteries with H_2_O‐H_3_PO_4_ electrolyte exhibit complete capacity degradation after 2 000 cycles, due to rapid decomposition of electrolyte at high potential (Figure 2i).

### 2.3 Proton Storage Mechanism

**Figure 3:**
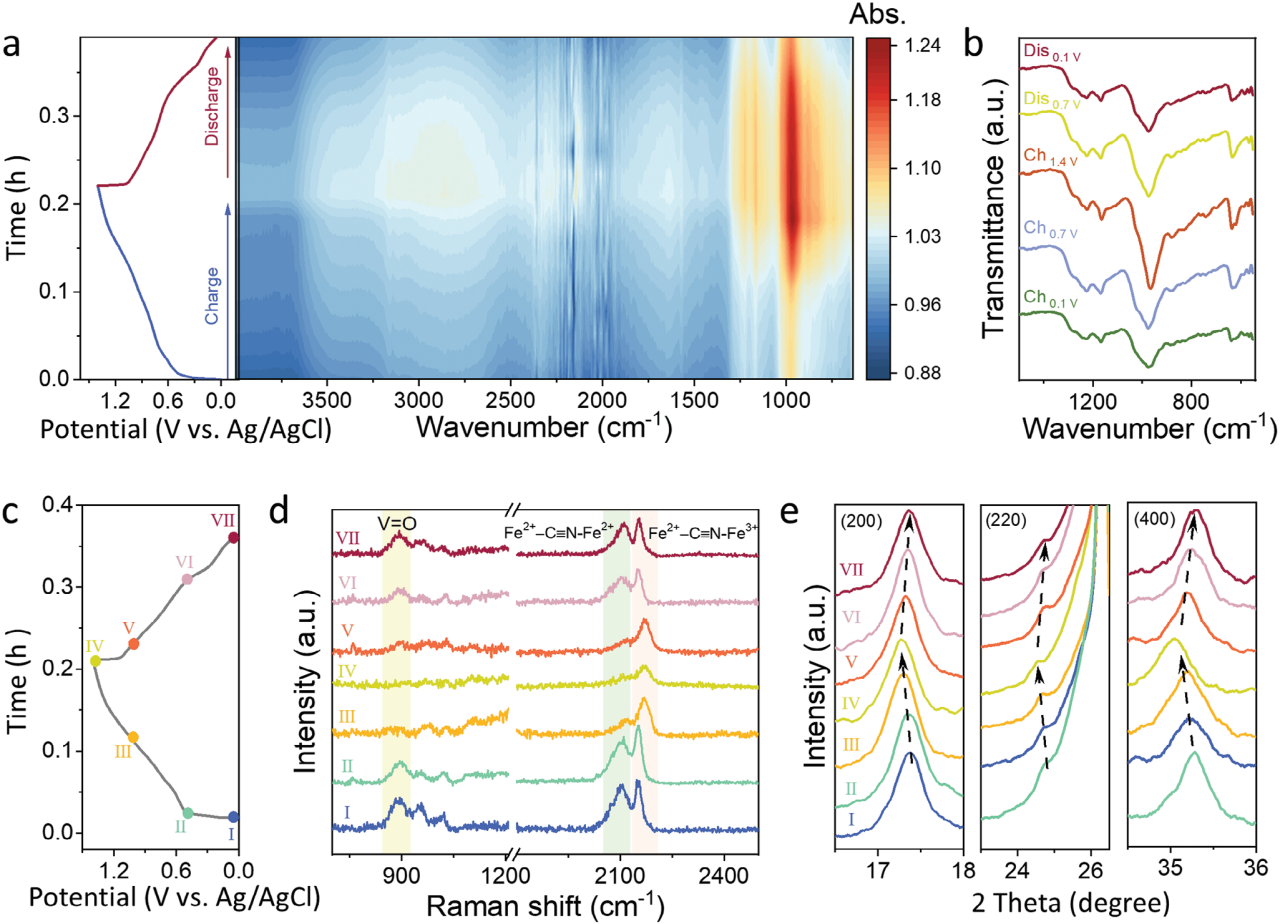
Proton storage mechanism. a,b) In situ FTIR spectra of H‐VHCF in EMImOTf‐H_3_PO_4_. c) Time‐potential curves at 1 A g^−1^, d) ex situ Raman spectra, and e) ex situ XRD profiles of H‐VHCF in EMImOTf‐H_3_PO_4_ electrolyte.

A series of in situ and ex situ tests were conducted to investigate the proton storage process of H‐VHCF cathode in EMImOTf‐H_3_PO_4_ electrolyte. In situ FT‐IR was conducted to monitor the structural change of the electrode (**Figure** **3**a,b; Figure S16, Supporting Information). During the charging process, the O─H bond vibration peaks at 3480 and 1660 cm^−1^ strengthen, while they weaken during the discharging process. This indicates that protons reversibly migrate between the electrolyte and the electrode material during charging and discharging, and the hydrogen bond strength at the electrode–electrolyte interface undergoes reversible changes, demonstrating good reversibility and stable proton storage and release characteristics (Figure 3a; Figure S16, Supporting Information).^[^ ^28^ ^]^ Prominent FT‐IR peaks at 970 and 635 cm^−1^ correspond to the V═O and Fe─C functional groups of H‐VHCF, respectively (Figure 3a,b).^[^ ^29^ ^]^ As the charging process progresses, the intensities of these two peaks gradually increase due to proton extraction, which leads to an increase in the oxidation states of vanadium and iron. Upon the discharging, the intensity of these two peaks slowly decreases, suggesting that the reversible insertion of proton into H‐VHCF results in the reduction of vanadium and iron. The periodic variation of the V═O peak and Fe─C peak intensities validate the role of dual active redox sites (V and Fe). Ex situ Raman spectra of H‐VHCF cathode in different charged/discharged states at 1 A g^−1^ were further characterized (Figure 3c,d; Figure S17, Supporting Information). During the proton extraction process (stage I to IV), the disappearance of the peak at 896 cm^−1^ corresponding to V═O group of H‐VHCF indicates the deformation of V═O bond.^[^ ^30^ ^]^ In the stage of IV to VII, the peak reappears, demonstrating the structure stability of H‐VHCF during H^+^ insertion and extraction. In addition, the peak at 2107 cm^−1^ associated with Fe^2+^‐C≡N‐Fe^2+^ gradually decreases and even disappears from states I to IV, while the peak at 2153 cm^−1^ ascribed to Fe^2+^‐C≡N‐Fe^3+^ experiences a blue‐shift. And a reverse tendency of the two peaks is observed from states IV to VII (Figure S17, Supporting Information).^[^ ^31^, ^32^ ^]^ This reversible change reflect the oxidation of Fe^2+^ to Fe^3+^ during the charging process and reduction of Fe^3+^ to Fe^2+^ during the discharging process, further underscoring the role of Fe as another redox active site. Ex situ XRD patterns of the electrode reveal neither disappearance of existing diffraction peaks nor the appearance of new peaks during the charging‐discharging process, suggesting no phase change occurs (Figure S18, Supporting Information). Nevertheless, small reversible shift of (200), (220), and (400) diffraction peaks observed in the process of charging and discharging demonstrate the lattice expansion and contraction due to proton extraction and insertion, respectively (Figure 3e). The Rietveld refinement of XRD patterns show that the lattice parameters of H‐VHCF are 10.1 Å (*R_wp_* = 6.7%, χ^2^ = 4.8%), 11.8 Å (*R_wp_* = 9.7%, χ^2^ = 5.4%), and 10.4 Å (*R_wp_* = 5.9%, χ^2^ = 4.6%) in the states of I, V, and VII, respectively (Figure S19, Supporting Information), which further indicates that the lattice parameter increases after proton extraction and decrease after proton insertion. High oxidation states of Fe and V lead to the extension of Fe─C and V─N bonds, increasing the overall distance between V and Fe sites.^[^ ^33^ ^]^ These characterizations demonstrate that the H‐VHCF electrode maintains excellent structural stability throughout the charging and discharging process, which could be primarily attribute to the reduced side reactions in an EMImOTf‐H_3_PO_4_ electrolyte. The stability of both electrolyte and electrode are critical to ensure the high performance and long‐cycle stability of proton batteries.

### 2.4 Electrochemical Performance of Full Proton Batteries

**Figure 4:**
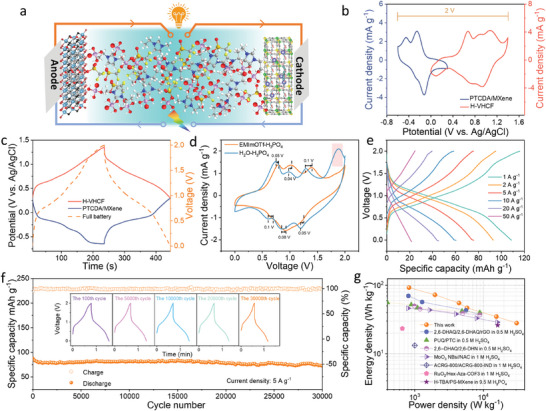
Electrochemical performance of PTCDA/MXene//EMImOTf‐H_3_PO_4_//H‐VHCF full proton battery. a) Schematic diagram of the full proton battery. b) CV curves at 5 mV s^−1^ and c) dynamic GCD curves of H‐VHCF, PTCDA/MXene, and full proton battery. d) CV curves in various electrolytes. e) GCD curves at various current densities. f) Long‐term cycling performance. g) Ragone plots of different battery systems.

To explore the practical application potential of EMImOTf‐H_3_PO_4_ as an electrolyte for proton batteries, an asymmetric proton battery was constructed with H‐VHCF as the cathode, and perylene‐3,4,9,10‐tetracarboxylic dianhydride (PTCDA)/MXene composite as the anode (**Figure** **4**a). The characterization and electrochemical performance of PTCDA/MXene are shown in Figures S20–S22 (Supporting Information), respectively. According to the CV curves of H‐VHCF (0 ∼ 1.4 V) and PTCDA/MXene (−0.6 ∼ 0.3 V) at 5 mV s^−1^, the PTCDA/MXene//EMImOTf‐H_3_PO_4_//H‐VHCF full proton batteries exhibit a broad operating voltage window of 0 ∼ 2 V (Figure 4b), which surpasses the reported operating voltages of all currently available proton batteries at room temperature. The potential changes of cathode and anode during the operation of full proton batteries were monitored in real‐time by introducing an additional Ag/AgCl reference electrode. As shown in Figure 4c, the actual operating potential range for H‐VHCF is 0.3 ∼ 1.4 V, while that for PTCDA/MXene is −0.6 ∼ 0.3 V. In the overlapping potential range (0 ∼ 0.3 V, Figure 4b), the electrochemical reaction is primarily dominated by PTCDA/MXene due to its lower polarization. The comparison of CV curves for PTCDA/MXene//EMImOTf‐H_3_PO_4_//H‐VHCF at 5 mV s^−1^ reveals that the potentials of redox peaks based on EMImOTf‐H_3_PO_4_ are higher than those based on H_2_O‐H_3_PO_4_, indicating the suitability of the EMImOTf‐H_3_PO_4_ electrolyte for high voltage applications. In addition, the CV curve in H_2_O‐H_3_PO_4_ electrolyte shows an irreversible oxidation peak at 1.9 V caused by water decomposition (Figure 4d).^[^ ^34^ ^]^ The GCD profiles at various current densities are presented in Figure 4e. The PTCDA/MXene//EMImOTf‐H_3_PO_4_//H‐VHCF batteries display the discharge capacities of 113.5, 92.8, 75.8, 60.5, 45.2, and 22.7 mAh g^−1^ at 1, 2, 5, 10, 20, and 50 A g^−1^, respectively. As the current density gradually is returned to 1 A g^−1^, the capacity recovers to 95.6% of its initial value, demonstrating the resilience to high current shock (Figure S23, Supporting Information). Moreover, the PTCDA/MXene//EMImOTf‐H_3_PO_4_//H‐VHCF demonstrate excellent stability with a capacity retention of 99.8% after 15 000 cycles and 86.1% after 30 000 cycles at a current density of 5 A g^−1^, indicating its potential for long‐term application (Figure 4f). The Ragone plot of the proton batteries, which features the highest operating voltage of 2 V compared to currently available proton batteries, shows a maximum energy density of 87.5 Wh kg^−1^ and a maximum power density of 30.6 kW kg^−1^. These values are not only twice those of electrochemical capacitors but also surpass the most of reported proton batteries (Figure 4g; Table S2, Supporting Information). The outstanding performance indicates the significant potential of EMImOTf‐H_3_PO_4_ to achieve the high energy storage and rapid power delivery of proton batteries, broadening the application field of proton batteries.

**Figure 5:**
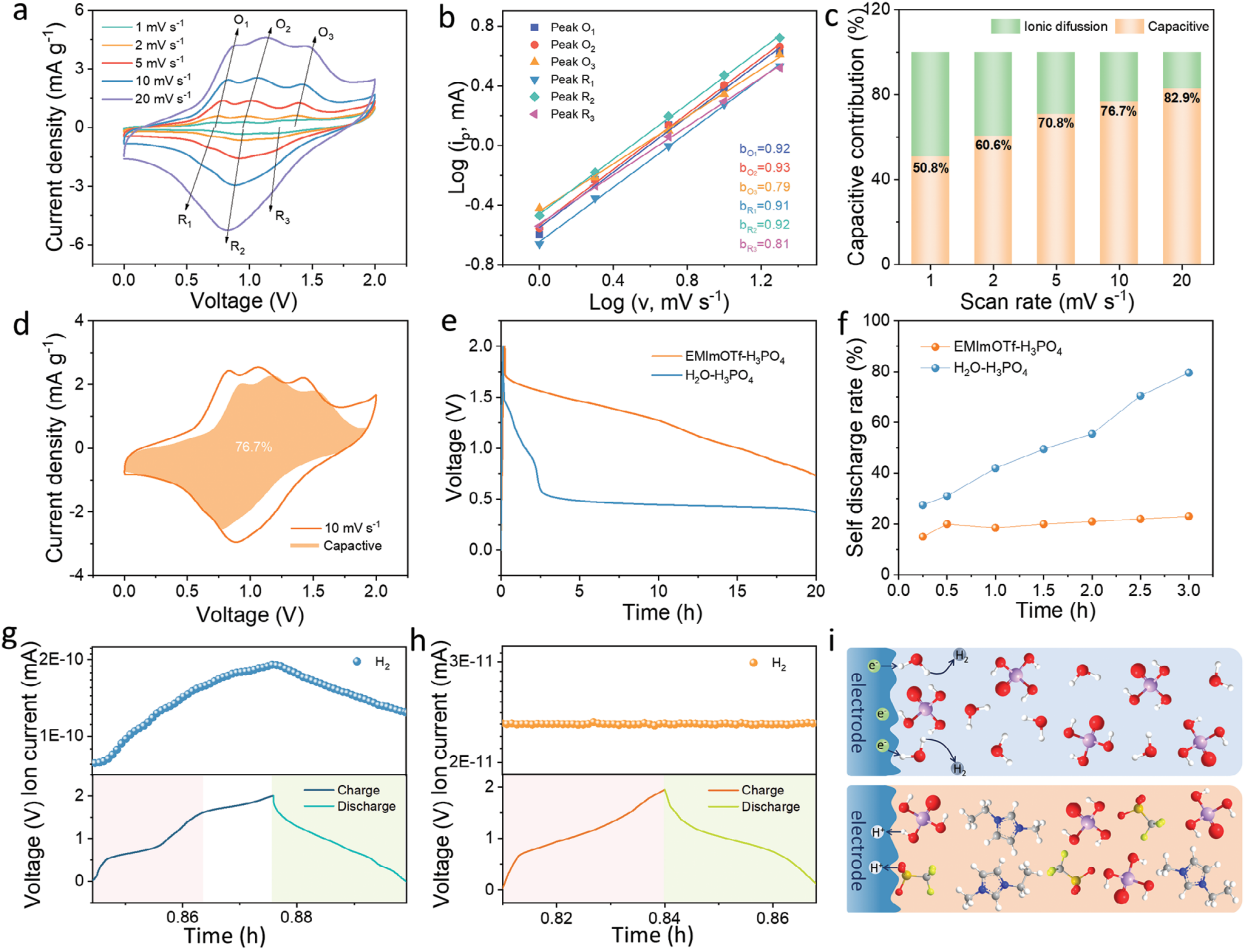
Electrochemical kinetics of PTCDA/MXene//EMImOTf‐H_3_PO_4_//H‐VHCF. a) CV curves at different scan rates. b) Logarithmic plots of peak current (*i*) versus scan rate (*v*). c) Capacitive contributions at different scan rates. d) CV curves with the capacitive contribution at 10 mV s^−1^. e) Voltage–time curves for 20 h rest. f) Self‐discharge rates at various rest times. Voltage–time curves (lower panels) and ion current‐time curves (upper panels) of full proton batteries in g) H_2_O‐H_3_PO_4_ and h) EMImOTf‐H_3_PO_4_. i) Schematic diagram of H_2_ evolution for H_2_O‐H_3_PO_4_ (upper) and EMImOTf‐H_3_PO_4_ (lower).

By analyzing the CV curves at different scan rates (**Figure** **5**a), the rapid reactive kinetics of H^+^ insertion/extraction in the PTCDA/MXene//EMImOTf‐H_3_PO_4_//H‐VHCF can be further elucidated. Based on the slopes of log (*i*) versus log (*v*) curves for the redox peaks (Figure 5b), the calculated *b* values of O_1_/R_1_, O_2_/R_2_, and O_3_/R_3_ are 0.92/0.91, 0.93/0.92, and 0.79/0.81, respectively, indicating the insertion/extraction of H^+^ are non‐diffusion controlled. As the scan rate increases, the capacitive contribution increases. The capacitive contribution reaches 76.7% at 10 mV s^−1^ (Figure 5c), and 82.9% at 20 mV s^−1^ (Figure 5d). Self‐discharging behavior is crucial in practical application but is often overlooked. PTCDA/MXene//EMImOTf‐H_3_PO_4_//H‐VHCF with non‐aqueous EMImOTf‐H_3_PO_4_ electrolyte exhibits a lower self‐discharge rate compared to one in traditional H_2_O‐H_3_PO_4_ electrolyte (Figure 5e). In fact, the self‐discharge rate of the batteries in H_2_O‐H_3_PO_4_ reaches 80% after 3 h of rest, whereas only 20% in EMImOTf‐H_3_PO_4_ (Figure 5f). The low self‐discharge rate reflects the electrochemical stability of EMImOTf‐H_3_PO_4_ electrolyte. It effectively suppresses side reactions and reduces energy loss, thereby enhancing the cycle life and overall performance of battery. As a cutting‐edge methodology, differential electrochemical mass spectrometry (DEMS) was further adopted to investigate the electrolyte stability by continuously tracking the gas evolution during the electrochemical process, then enabling the assessment of electrolyte degradation or changes.^[^ ^35^ ^]^ Figure 5g,h demonstrates the voltage–time profiles (lower panels) and corresponding ion current–time curves of H_2_ (upper panels). The signal of ion current is proportional to the gas evolution rate. As illustrated in Figure 5g, the ion current signal of H_2_ increases with the rising voltage, indicating the continuous decomposition of H_2_O‐H_3_PO_4_ electrolyte during the charging process. This behavior is likely related to the breakdown of water molecules and the generation of H_2_. The charging plateau at 1.7 V in the lower panel of Figure 5g also reflects this process. In contrast, the full proton battery in EMImOTf‐H_3_PO_4_ electrolyte exhibits no changes in the ion current of H_2_ during the charging and discharging cycles, demonstrating the superior stability (Figure 5h). Water molecules on the interface of electrode and electrolyte can readily capture electrons leading to hydrogen production and reducing the stability of the aqueous acid electrolyte. In contrast, the EMImOTf‐H_3_PO_4_ electrolyte minimizes active water, effectively preventing self‐decomposition under high voltage (Figure 5i).^[^ ^36^, ^37^ ^]^ This mechanism ensures the durability of the ILs solvent electrolyte under high voltage and the overall safety of the battery system.

### 2.5 The Performance of PTCDA/MXene//EMImOTf‐H_3_PO_4_//H‐VHCF Proton Batteries at Wide Temperature Range

**Figure 6:**
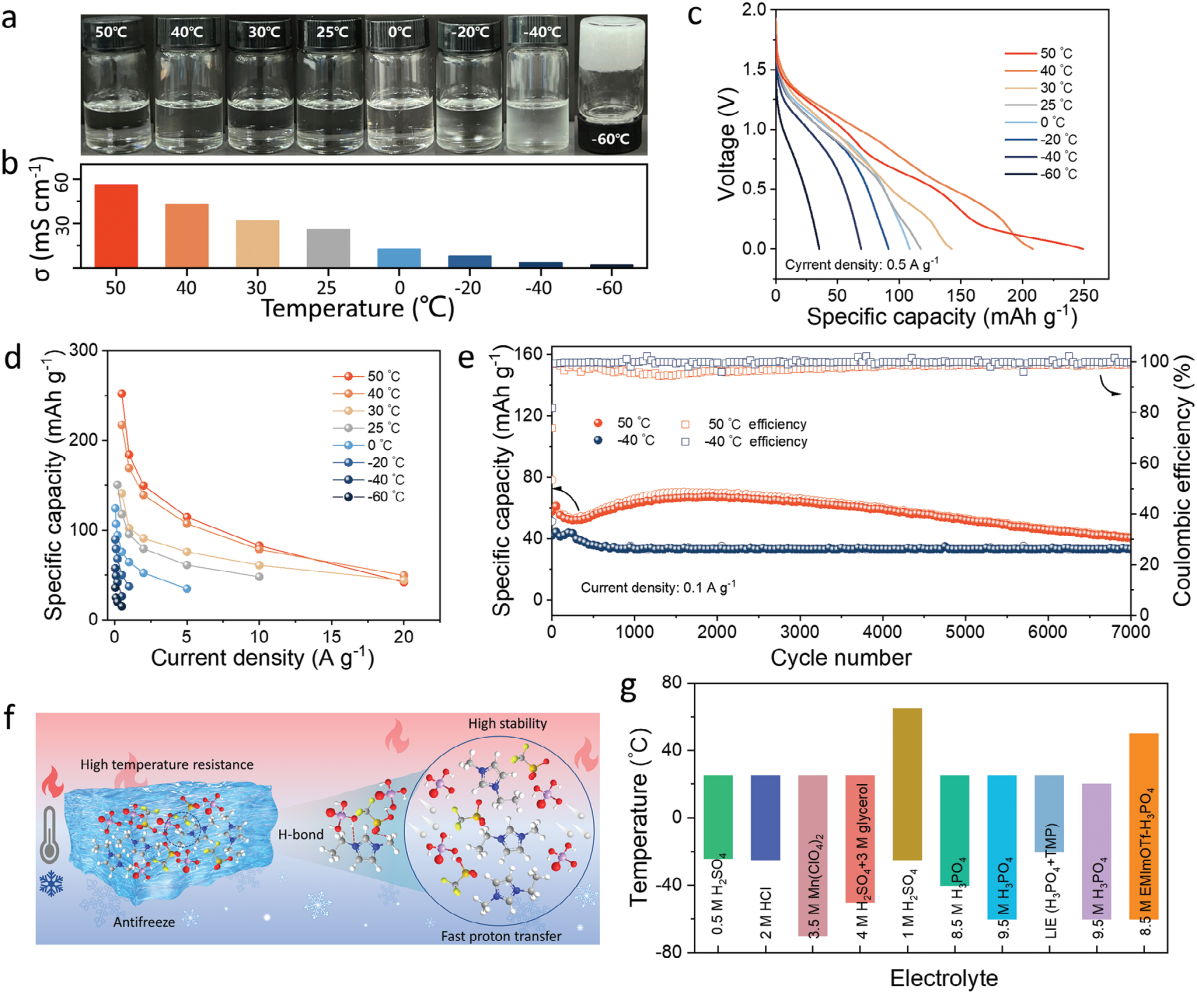
The PTCDA/MXene//EMImOTf‐H_3_PO_4_//H‐VHCF full proton battery operates within a wide temperature range from 50 to −60 °C. a) Digital photos and b) ion conductivities of EMImOTf‐H_3_PO_4_ electrolyte at various temperatures. c) Discharging curves at 0.5 A g^−1^ and d) rate performance at different temperatures. e) Long‐term cycling performance at 50 and −60 °C. f) Illustration for the high stability of EMImOTf‐H_3_PO_4_ electrolyte at a wide temperature range. g) Comparison of the operating temperature range with other proton batteries.

The intricate hydrogen bonding network significantly inhibits the decomposition of the EMImOTf‐H_3_PO_4_ electrolyte at high temperature and prevents the crystallization at low temperature, therefore ensuring the stability and functionality across a wide temperature range. The physical states and ion conductivities at 50 ∼ −60 °C are shown in **Figure** **6**a,b. EMImOTf‐H_3_PO_4_ remains a stable liquid state from 50 to −40 °C, until freezes at −60 °C. Additionally, the ion conductivity of EMImOTf‐H_3_PO_4_ declines as the temperature decreases (Figure 6b). The peaks in the Raman spectra progressively sharpen from 50 to −60 °C due to the reduction in molecular thermal motion as the temperature decreases (Figure S24, Supporting Information). The noticeable sharp transition in the Raman spectra at −60 °C is attributed to the freezing of electrolyte, as confirmed by the digital photographs during the Raman tests (Figure S25, Supporting Information). The CV tests of PTCDA/MXene//EMImOTf‐H_3_PO_4_//H‐VHCF reveal that the operating voltage window is expanded from 0–2 to 0–2.6 V with the decreasing of the operating temperature from 25 to −60 °C (Figure S26, Supporting Information). This improvement may be attributed to the reduce rate of side reactions at low temperatures, which enhance the stability of the system for operation at high voltages. At 0.5 A g^−1^, the PTCDA/MXene//EMImOTf‐H_3_PO_4_//H‐VHCF exhibits the discharge capacities of 249.8 mAh g^−1^ at 50 °C, 208.4 mAh g^−1^ at 40 °C, 142.7 mAh g^−1^ at 30 °C, and 117.3 mAh g^−1^ at 25 °C. At low temperatures, the capacities are 108.6 mAh g^−1^ at 0 °C, 91.4 mAh g^−1^ at −20 °C, 69.2 mAh g^−1^ at −40 °C, and remain at 45.2 mAh g^−1^ at −60 °C (Figure 6c). These results indicate that the battery demonstrates good discharge performance across a wide temperature range, from elevated temperatures to cold. Furthermore, at −40 °C, the PTCDA/MXene//EMImOTf‐H_3_PO_4_//H‐VHCF demonstrates a specific capacity of 57.4 mAh g^−1^ at 0.05 A g^−1^, retaining 45.7% capacity at 0.5 A g^−1^. At −60 °C, when the electrolyte is frozen, the specific capacity is 56 mAh g^−1^ at 0.05 A g^−1^, decreasing to 30 mAh g^−1^ at 0.2 A g^−1^ (Figure 6d). These results suggest that protons remain transportable in the solid state, consistent with the characteristics of the Grotthuss mechanism. Long‐term cycling tests at 0.1 A g^−1^ display that the capacity retention of full battery is 70.2% at 50 °C and 76.6% at −60 °C after 7000 cycles (Figure 6e). The superior electrochemical performance of the full proton batteries across a broad temperature range is attributed to the stable hydrogen bonding network within the EMImOTf‐H_3_PO_4_ electrolyte. This network effectively prevents the electrolyte degradation at elevated temperature and inhibits the freeze at low temperature, thereby ensuring the stability and efficiency of proton battery under diverse environmental conditions (Figure 6f). The operational temperature range of the PTCDA/MXene//EMImOTf‐H_3_PO_4_//H‐VHCF system spans from 50 to −60 °C, covering a total range of 110 °C, positioning it as one of the broadest temperature ranges among the known proton batteries (Figure 6g; Table S3, Supporting Information). The pouch cell of PTCDA/MXene//EMImOTf‐H_3_PO_4_//H‐VHCF can power a timer at 50 °C (Figure S27a, Supporting Information), 25 °C (Figure S27b, Supporting Information), and −60 °C (Figure S27c, Supporting Information), effectively demonstrating its practicality across a broad temperature range. The ability of proton batteries to operate across a wide temperature range provides reliable support for energy storage and conversion under diverse climatic conditions. The assembled pouch cell demonstrates excellent safety characteristics, further confirming its potential for practical applications. (Figure S28, Supporting Information).

## 3 Conclusion

In summary, this study introduces a promising strategy for developing non‐aqueous IL electrolytes, which significantly expands the electrochemical stability window and enhances the cycling stability of proton battery. The employment of the IL solvent (EMImOTf) effectively mitigates the hydrogen/oxygen evolution and reduces the corrosion of electrode. A series of characterization techniques, including NMR, FT‐IR, and Raman spectroscopy, reveal the multiple and enhanced hydrogen bond interactions between EMImOTf and H_3_PO_4_ molecules. MD simulations indicate that the EMImOTf‐H_3_PO_4_ electrolyte features a complex hydrogen bonding network with multiple interactions. Furthermore, investigations into the electrochemical proton storage of the H‐VHCF cathode in EMImOTf‐H_3_PO_4_ demonstrate a remarkable 126% increase in CE compared to conventional acidic aqueous electrolytes. In situ FTIR, ex situ Raman, and ex situ XRD analyses confirm the reversible insertion and extraction of protons during the charging and discharging processes, highlighting the stability of the IL electrolyte. The proton transport facilitated by the hydrogen‐bonding network in the EMImOTf‐H_3_PO_4_ electrolyte, coupled with the dual redox reactions of vanadium and iron in the H‐VHCF cathode, significantly improves the specific capacity. The PTCDA/MXene//EMImOTf‐H_3_PO_4_//H‐VHCF full proton battery exhibits remarkable performance, retaining 86.1% of its initial capacity after 30 000 cycles at a high operating voltage of 2 V, which exceeds the currently reported voltage value. At room temperature, the battery achieves an energy density of 87.5 Wh kg^−1^, and a power density of 30.6 kW kg^−1^, and it demonstrates stable performance over a broad temperature range from 50 to −60 °C. This research enables the development of high‐voltage electrolytes for proton batteries in all‐climate smart grid systems, addressing needs for efficient frequency regulation, emergency power support, and flexible power scheduling.

## Conflict of Interest

The authors declare no conflict of interest.

## Supporting information



Supporting Information

## Data Availability

The data that support the findings of this study are available from the corresponding author upon reasonable request.
