# Supplementary material for: Ionic Liquid‐Based Electrolyte with Multiple Hydrogen Bonding Network Enabling High‐Voltage Stable Proton Batteries Across Wide Temperature Range
Source: Adv Sci (Weinh). 2025 Feb 14;12(14):2416931. doi: 10.1002/advs.202416931 (PMC11984899; doi:10.1002/advs.202416931)
Supplement: Supplementary file 1 — Supporting Information [file ADVS-12-2416931-s001.docx]

**Supporting Information**

**Ionic Liquid-based Electrolyte with Multiple Hydrogen Bonding Network Enabling High-Voltage Stable Proton Batteries across Wide Temperature Range**

*Xiaoyu Dong^1^, Zhiwei Li^1^, Hai Xu^1^, Zhiyuan Wu^1^, Fanhao Meng^1^, Shuzhi Liu^2^, Hui Dou^1^***, Xiaogang Zhang^1^**

^1^Jiangsu Key Laboratory of Materials and Technologies for Energy Storage Technology, College of Material Science and Technology, Nanjing University of Aeronautics and Astronautics, Nanjing, 210016, People’s Republic of China

^2^Chemical and Biomolecular Engineering, National University of Singapore, Singapore 117585, Singapore

All correspondence and request for materials should be addressed to: [dh_msc@nuaa.edu.cn](mailto:dh_msc@nuaa.edu.cn) (Prof. Hui Dou), [azhangxg@nuaa.edu.cn](mailto:azhangxg@nuaa.edu.cn) (Prof. Xiaogang Zhang).

Keywords: Proton batteries, Ionic liquid electrolyte, High-voltage, multi-level hydrogen bonding network

**Experimental Section**

*Synthesis of protonated vanadium hexacyanoferrate (H-VHCF):* To synthesize H-VHCF, 50 mL of HCl was diluted to 1.5 times its original volume. Then, 22.1 mmol of V_2_O_5_ was added to form a yellow suspension. Next, 9.6 mmol of glycerol was introduced under constant stirring at 60℃ until a clear blue solution formed. 9.375 mL of above solution was diluted to 50 mL to prepare solution A. Separately, 3.6 mmol of K_3_Fe(CN)_6_ was dissolved in deionized water to prepare 50 mL of solution B. Under magnetic stirring at 60℃, solution B was added to solution A and stirred continuously for 9 hours. The mixture was then cooled to room temperature, and the precipitate was collected by centrifugation. The green precipitate was air-dried at 80℃ overnight to yield VHCF. For chemical reduction, 1 g of VHCF was dispersed in 20 mL of deionized water (oxygen was removed using nitrogen). Subsequently, 10 mL of hydrazine hydrate solution (0.05 M) was added, and the mixture was stirred for 10 minutes before centrifugation. The H-VHCF product was obtained by drying in a vacuum oven at 40℃.

*Preparation of MXene:* Few-layer Ti_3_C_2_Tx (MXene) was synthesized using Ti_3_AlC_2_ as a precursor. First, 2 g of LiF was added to 20 mL of HCl. Then, 1 g of Ti_3_AlC_2_ was added to the solution, and the mixture was reacted at 50℃ for 48 hours. After reaction, the mixture was centrifuged to wash until a neutral pH was reached. The product was then re-dispersed in deionized water and subjected to ultrasonic treatment for 1 hour. The dark supernatant was collected, yielding few-layer Ti_3_C_2_Tx MXene.

*Synthesis of perylene-3,4,9,10-tetracarboxylic dianhydride (PTCDA)/MXene:* 190 mg of PTCDA was dispersed in 80 mL of deionized water and ultrasonicated for 20 minutes. Subsequently, 10 mL of MXenes dispersion (1 mg/mL) was added to the PTCDA suspension, followed by an additional 20 minutes of ultrasonication. The mixture was then freeze-dried overnight to obtain the PTCDA/MXene composite.

*Electrolyte preparation:* To synthesize an 8.5 M EMImOTf-H_3_PO_4_ electrolyte, 5.6 ml of 98% H_3_PO_4_ was mixed with 4.4 ml of EMImOTf. For comparison, 5.6 ml of 98% H_3_PO_4_ was mixed with 4.4 ml of H_2_O to prepare an 8.5 M H_2_O-H_3_PO_4_ electrolyte.

*Characterization:* The microstructures and morphologies of materials were analyzed using scanning electron microscope (SEM, Hitachi S-4800), transmission electron microscope (TEM, FEI Talos F200X), and X-ray diffraction (XRD, Synchrotron BL13W1). Fourier Transform infrared (FT-IR) spectroscopy was recorded with a Bruker Vertex 70 spectrometer. Raman spectroscopy was performed using a Horiba Scientific with a 532 nm laser for excitation. ^1^H and ^31^P nuclear magnetic resonance (NMR) spectra were acquired on a BRUKER AVANCE 400 MHz instrument with DMSO-*d_6_* as a solvent.

*Electrode preparation and electrochemical tests:* The H-VHCF cathode was prepared by mixing 70 wt.% H-VHCF, 20 wt.% Ketjen black carbon, and 10 wt.% poly-vinylidene fluoride (PVDF) binder. The slurry was then coated onto carbon fiber paper and vacuum-dried overnight at 60 ℃. The PTCDA/MXene anode was prepared using the same method. Electrochemical performance of electrolyte was evaluated using a Swagelok cell, with a self-supporting activated carbon membrane as the counter electrode and an Ag/AgCl electrode containing 3 M KCl solution as the reference electrode. A Whatman filter paper served as the separator. Liner sweep voltammetry (LSV) and cyclic voltammetry (CV) were conducted with a CHI electrochemical workstation. Galvanostatic intermittent titration technique (GITT), rate capability, cycling stability, and self-discharge tests were performed using LAND test system. Gas evolution was assessed using differential electrochemical mass spectrometry (DEMS).

*Molecular Dynamics (MD) Simulation:* The partial charges of H_3_PO_4_ and EMImOTf molecules were calculated using Gaussian 16 with the 6-311g (d, p) basis set. The OPLS-AA force filed and Auxiliary Tools of Force Field (AuToFF) were employed to parameterize all atoms, including bond, angle, and dihedral parameters. Molecular dynamics (MD) simulations were conducted to study interaction in the mixed system, with 46 EMImOTf and 200 H_3_PO_4_ molecules randomly inserted into a cubic box with a side length of 6.0 nm. Simulations were perform using the GROMACS 2021. Initial energy minimization was conducted with the steepest descent method, applying periodic boundary conditions. The Leapfrog algorithm integrate the equations of motion in an NPT ensemble for 20 nm. Pressure was maintained at 1 bar using the Berendsen barostat, and the temperature was kept at 298.15 K using the V-rescale thermostat. The LINCS algorithm constrained bond lengths involving hydrogen, while electrostatic interactions were calculated using the Particle-Mesh-Ewald (PME) method with a cutoff of 1.0 nm used for short-range van der Waals interactions.

Ion diffusion calculation formula:

$$D=\frac{4}{\pi\tau}{(\frac{m_{B}V_{m}}{M_{B}S})}^{2}{(\frac{\Delta E_{s}}{{\Delta E}_{t}})}^{2}$$

Herein, $\tau$ denotes the relaxation time (s), $M_{B}$, $V_{m}$ , and $M_{B}$ stand for the mass (g), molar volume (cm^3^ mol^-1^), and molar mass (g mol^-1^) of the active substance, respectively. The parameter $S$ (cm^2^) indicates the electrode area, while ${\Delta E}_{s}$ (V) characterizes the steady-state potential variation induced by current pulses, and ${\Delta E}_{t}$(V) describes the $iR$ drop occurring after the relaxation period.

The mass of cathode and anode matching is balanced according to the formula:

$$Q=C\times m\times\Delta E$$

$$\frac{m_{+}}{m_{-}}=\frac{C_{-}\times E_{-}}{C_{+}\times E_{-}}$$

Where $Q$ is the charge (mC), $m$, $C$, and $E$ represent the mass (mg), specific capacitance (mAh g^-1^), and the potential window (V) of the cathode and anode, respectively.

Energy density and power density of asymmetric proton batteries can be obtained as follows:

$$E=\frac{\int IVdt}{3.6m}$$

$$P=\frac{3600E}{\Delta t}$$

Where $E$ and $P$ are energy density (Wh kg^-1^) and power density (kW kg^-1^) respectively, $m$ is the total mass of active material in cathode and anode (mg), and $\Delta t$ is the discharge time (s).

The mean square displacement (MSD) of the molecules is linearly related to time. The diffusion coefficient of the molecules can be calculated using the following equation:

$$D=\lim_{t\to\infty} \frac{1}{6t}<{|r\left( t \right)-r\left( 0 \right)|}^{2}>$$

$<>$ denotes the average over all atoms in the group, t represents time, $r(t)$ and $r(0)$ denote the position of the molecules at time $t$ and $0$, respectively.


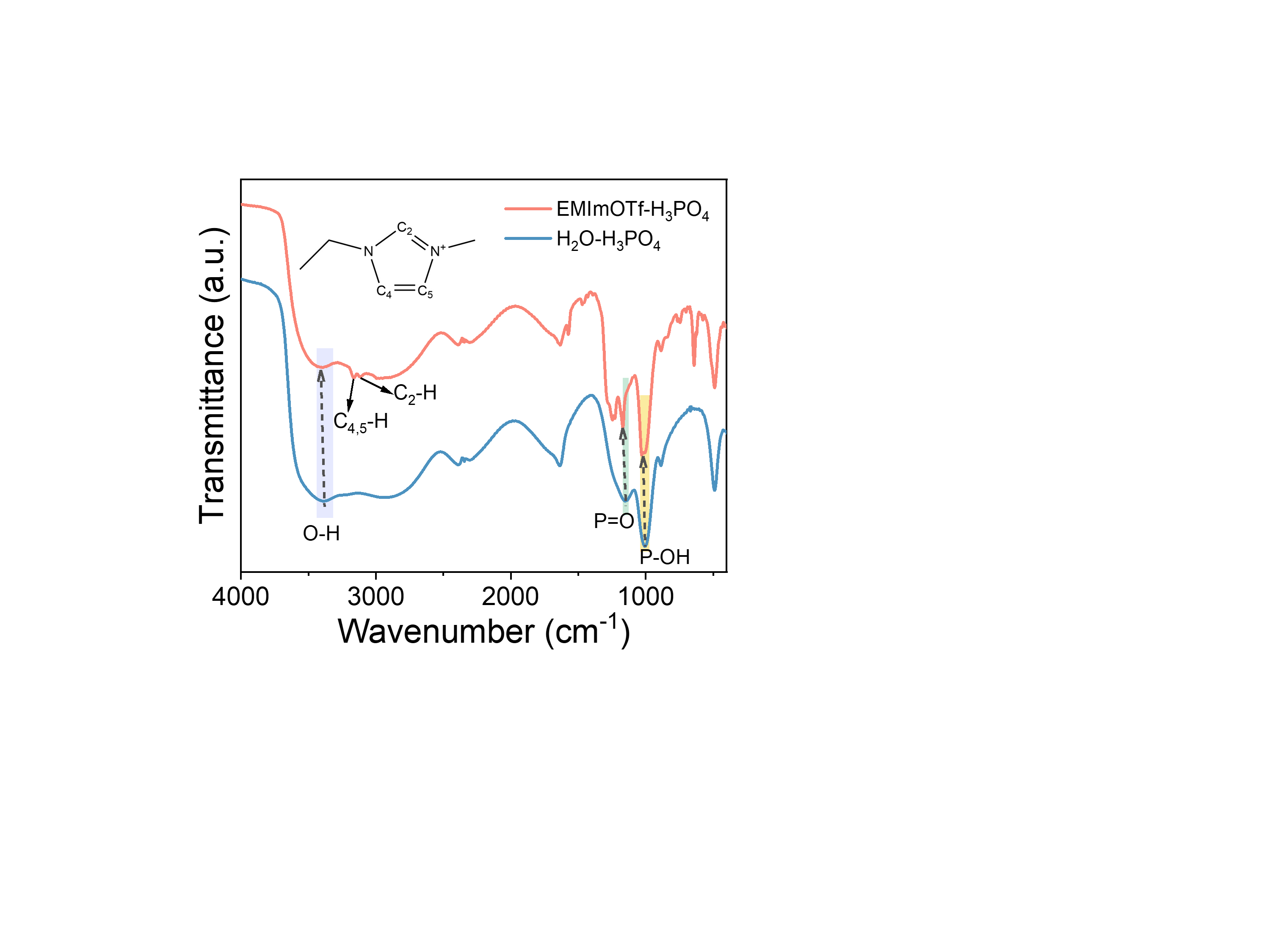


**Figure S1.** FTIR spectra of H_2_O-H_3_PO_4_ and EMImOTf-H_3_PO_4_ electrolytes.


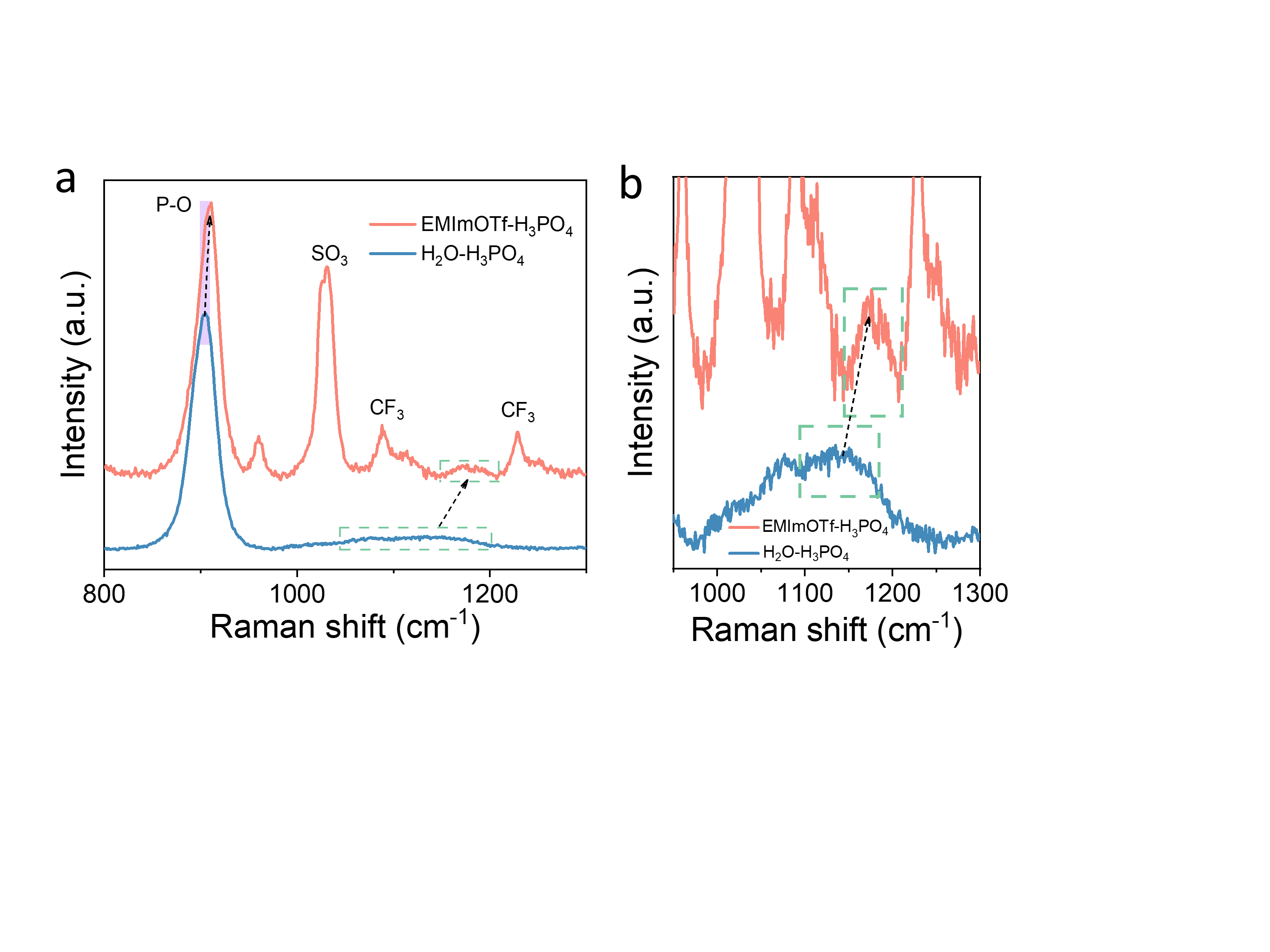


**Figure S2.** (a) Raman spectra of H_2_O-H_3_PO_4_ and EMImOTf- H_3_PO_4_ electrolytes, (b) and their close-ups at 950~ 2300 cm^-1^.


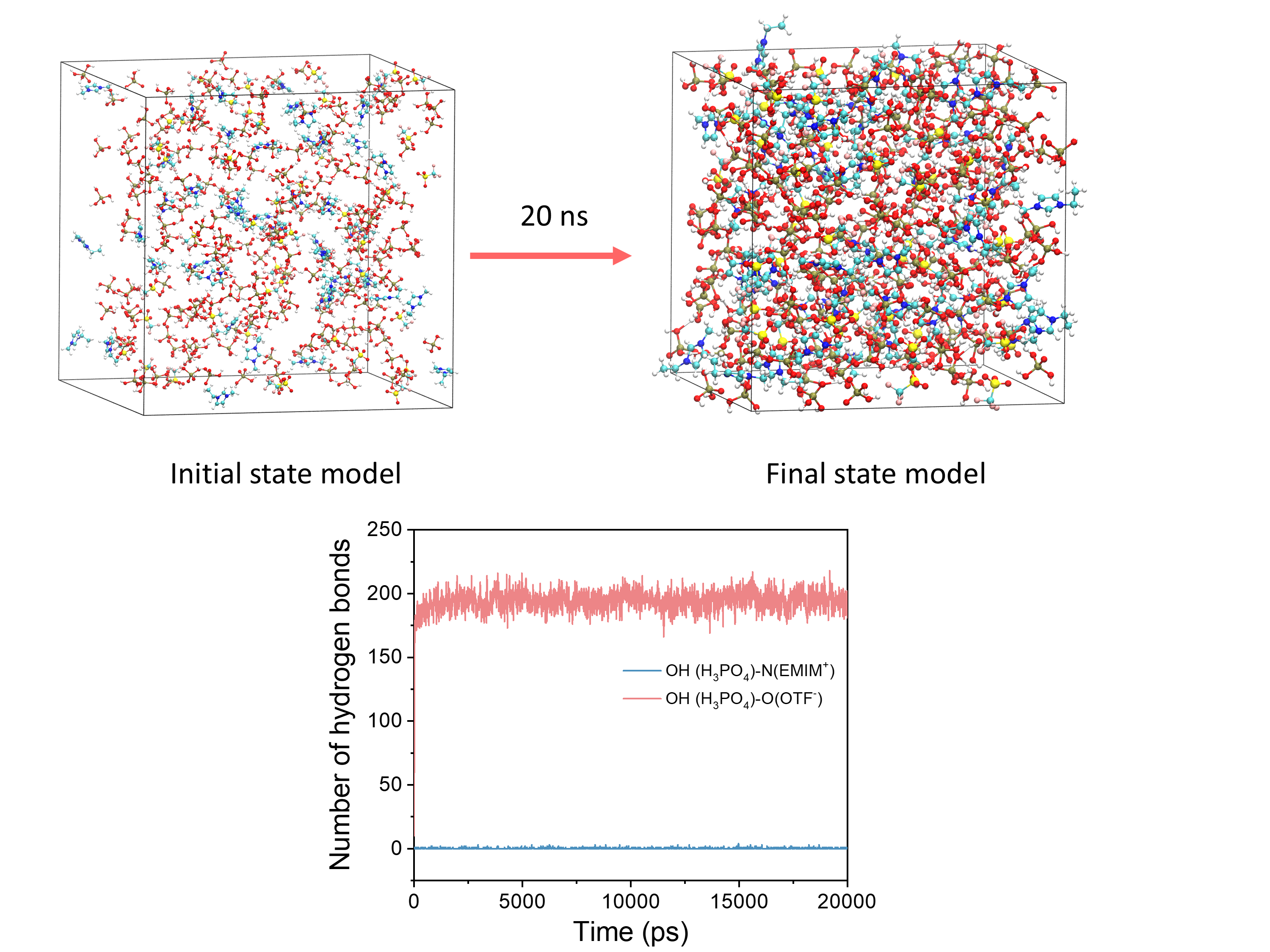


**Figure S3.** Model evolution diagram of EMImOTf-H_3_PO_4_.


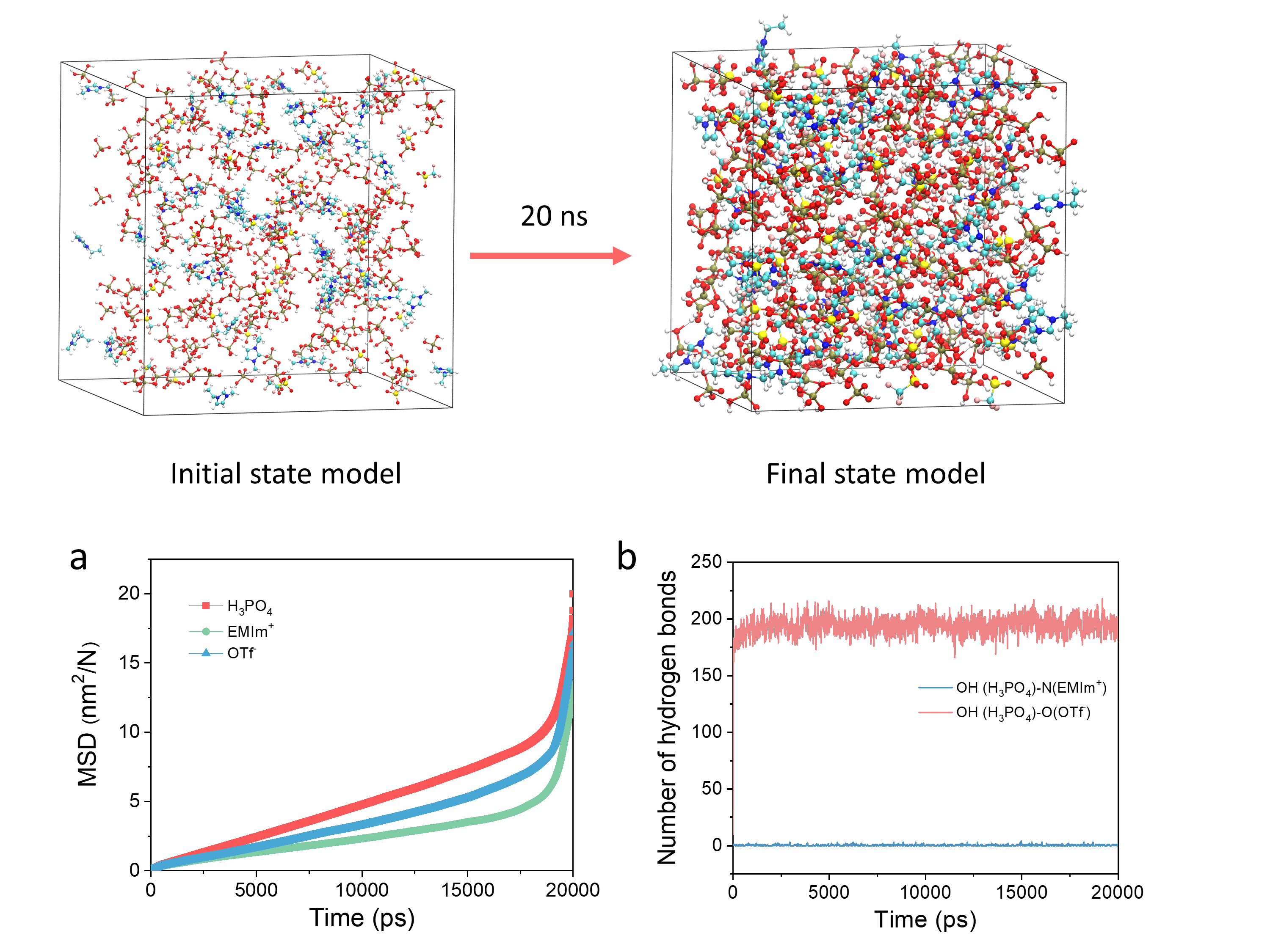


**Figure S4.** (a) The root means square displacement curves of H_3_PO_4_, EMIm^+^, and OTf^-^. (b) Numbers of hydrogen bond between H_3_PO_4_ and EMImOTf.


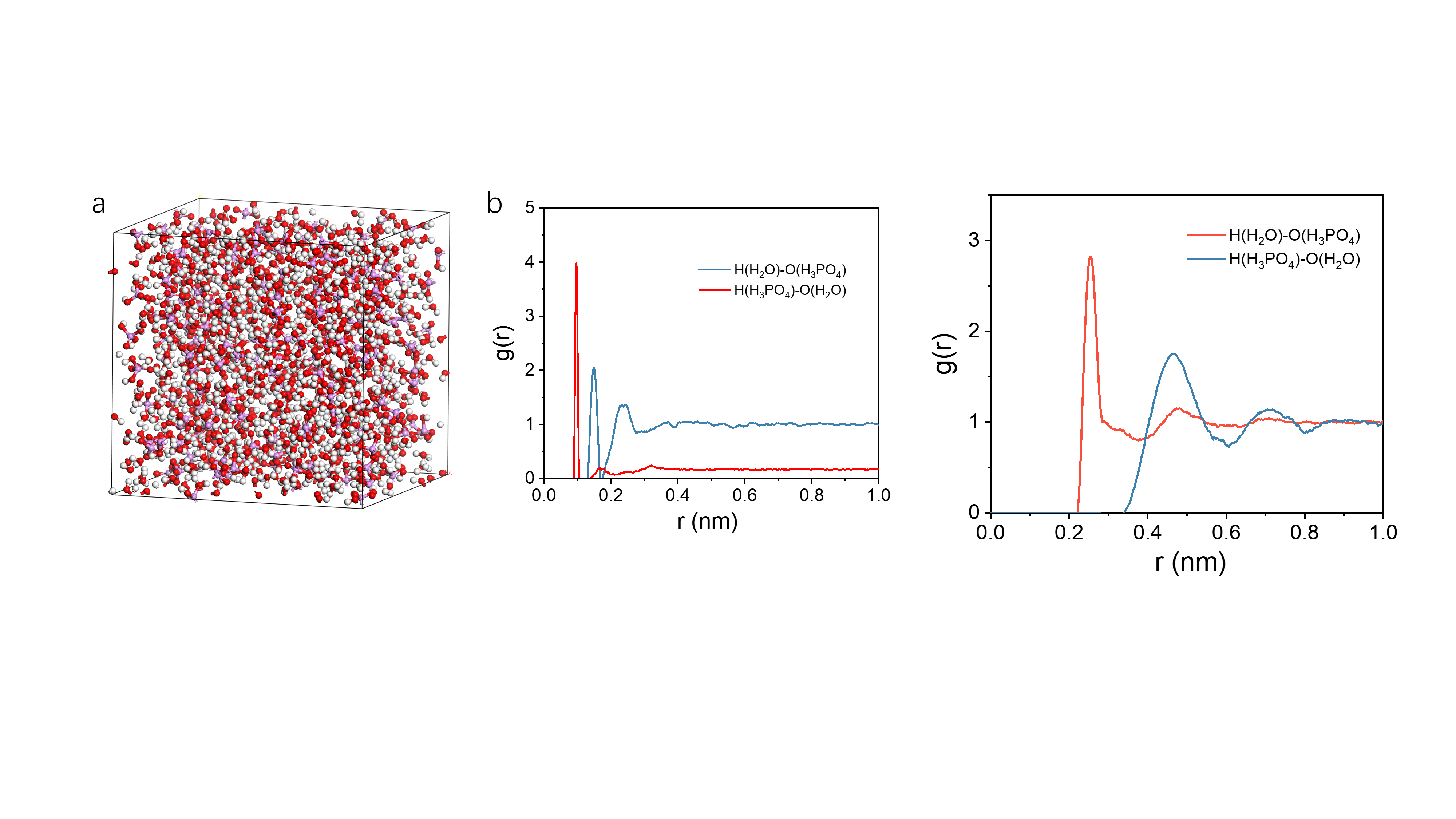


**Figure S5.** (a) The snapshot of H_2_O-H_3_PO_4_ electrolyte from MD simulation. (b) The RDFs for H (H_3_PO_4_)-N (H_2_O) and H (H_2_O)-O (H_3_PO_4_).


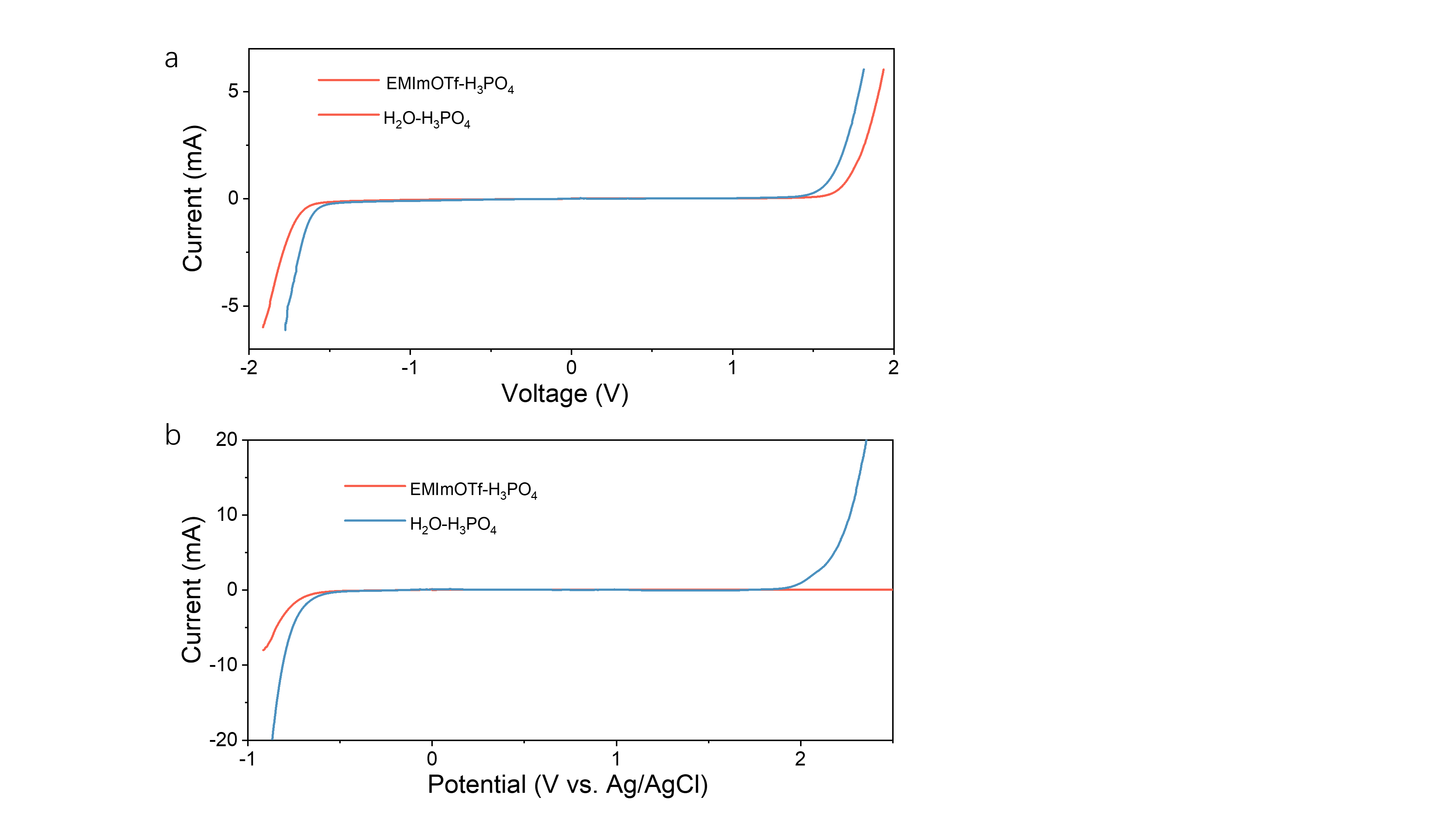


**Figure S6.** LSV profiles of different electrolytes in (a) stainless-steel symmetrical battery and (b) three-electrode system.


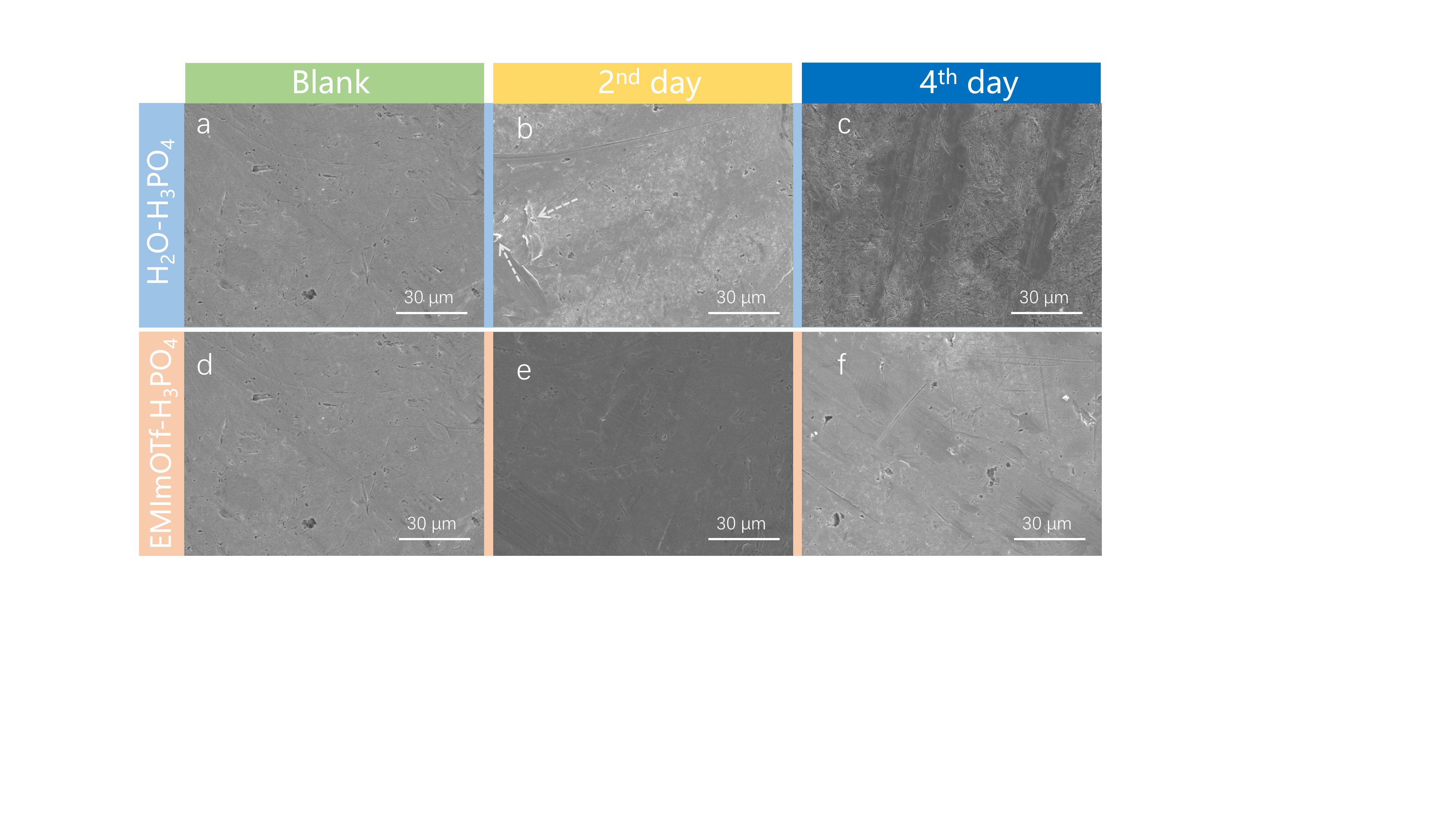


**Figure S7**. SEM images of stainless steel. (a) Initial state, after soaking for (b) 2 days and (c) 4 days in H_2_O-H_3_PO_4_. (d) Initial state, after soaking for (e) 2 days and (f) 4 days in EMImOTf-H_3_PO_4_.


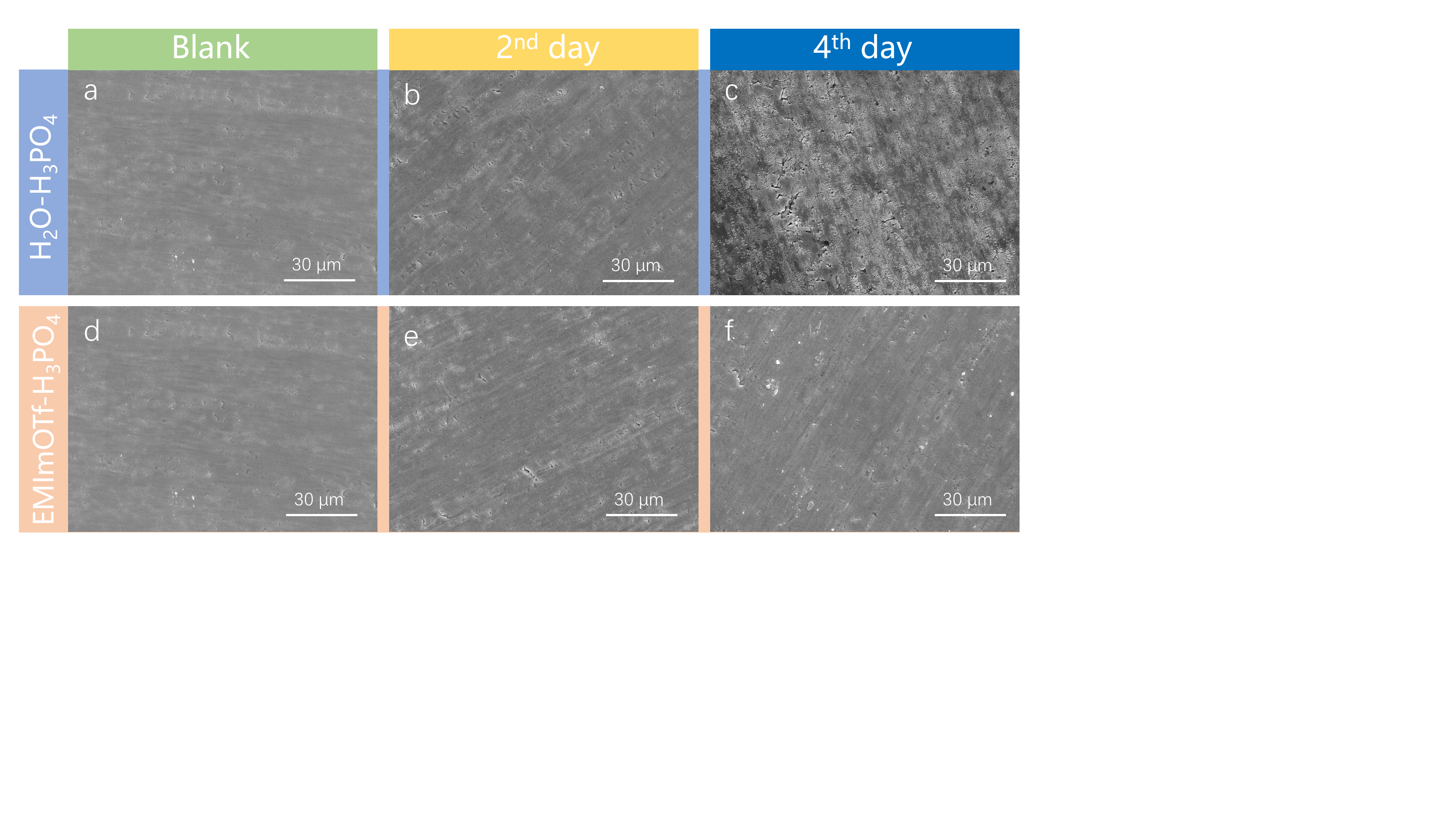


**Figure S8.** SEM images of Ti foil. (a) Initial state, after soaking for (b) 2 days and (c) 4 days in H_2_O-H_3_PO_4_. (a) Initial state, after soaking for (b) 2 days and (c) 4 days in EMImOTf-H_3_PO_4_.

In the H_2_O-H_3_PO_4_ electrolyte, stainless steel exhibits corrosion at defect sites after 2 days of immersion, which become notably severe after 4 days. In contrast, stainless steel in the EMImOtf-H_3_PO_4_ electrolyte shows no visible signs of corrosion throughout the 4 days (Figure S7). For titanium foil, no corrosion is observed in any electrolyte after 2 days of immersion. After 4 days, the H_2_O-H_3_PO_4_ electrolyte shows a corrosion trend, while the titanium foil in EMImOtf-H_3_PO_4_ electrolyte remains stable and corrosion-free (Figure S8). Compared to the H_2_O-H_3_PO_4_ electrolyte, EMImOtf-H_3_PO_4_ electrolyte exhibits superior anti-corrosion performance and excellent chemical stability for both stainless steel and titanium foil.


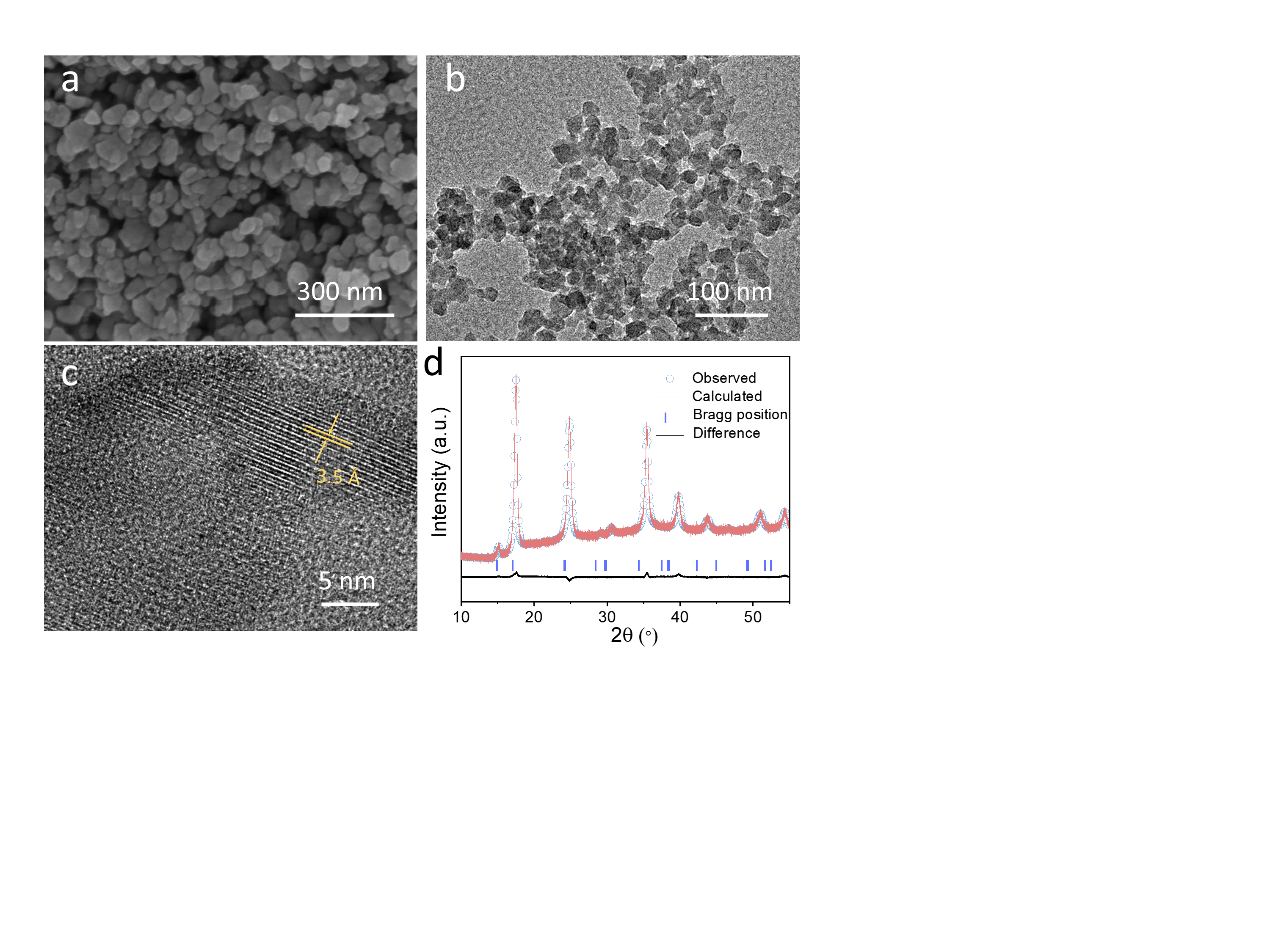


**Figure S9.** Characterization of H-VHCF. (a) SEM, (b) TEM, and (c) HRTEM images. (d) XRD Rietveld refinement profile.

Vanadium precursors were added to K_3_Fe(CN)_6_ solution and stirred at 60℃ to synthesize VHCF, which was then protonated to obtain H-VHCF. The microstructure of H-VHCF was characterized using SEM and TEM. The SEM and TEM (Figure S9a, Figure S9b, and FigureS9c) show that H-VHCF forms irregular clusters with an average diameter of approximately 20 nm, and the lattice spacing is 3.5 Å. The large surface area of these nanoparticles provides abundant actives sites for proton storage, thereby enhancing capacity. The XRD pattern (Figure S9d) of H-VHCF shows that all diffraction peaks correspond to the classical compound Cu[Fe(CN)_6_]_2/3_ (PDF No. 01-0239). Rietveld refinement analysis indicates that H-VHCF has a face-centered cubic structure with a space group of Fm3-m and a lattice parameter of 10.2 Å ($R_{wp}$= 8.4 %, $\chi^{2}$= 5.62 %).


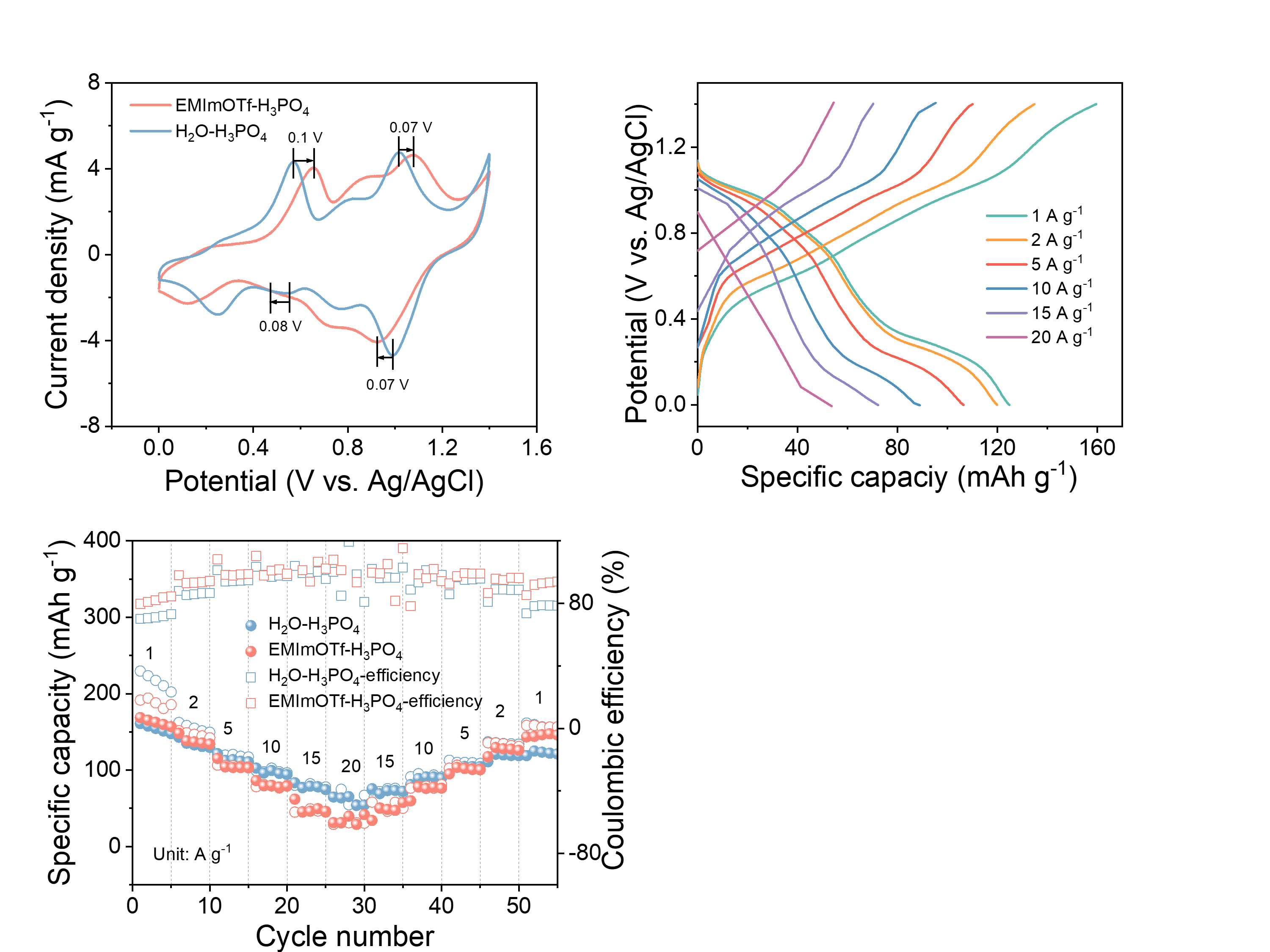


**Figure S10.** CV curves of H-VHCF cathode in EMImOTf-H_3_PO_4_ and H_2_O-H_3_PO_4_.


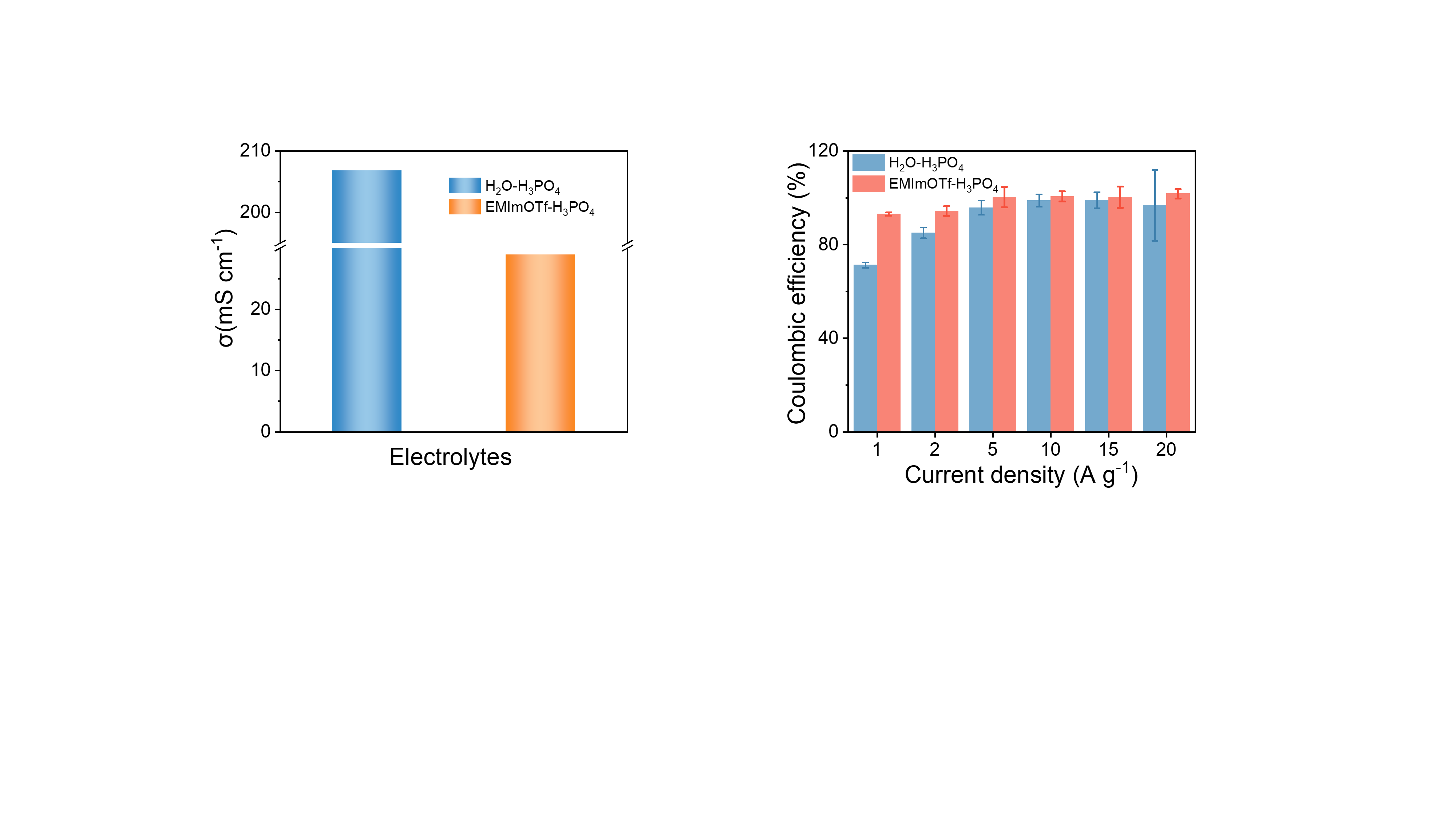


**Figure S11.** Coulombic efficiency statistics at different current densities of H_2_O-H_3_PO_4_ and EMImOTf-H_3_PO_4_ electrolytes. The error bars for EMImOTf-H_3_PO_4_ are 92.6±0.7% s.e.m. at 1 A g^-1^, 94.4±2.1% s.e.m. at 2 A g^-1^, 100.3±4.3% s.e.m. at 5 A g^-1^, 100.6±2.1% s.e.m. at 10 A g^-1^, 100.3±4.5% s.e.m. at 15 A g^-1^, 100.1±1.9% s.e.m. at 20 A g^-1^, where n=5. The error bars for H_2_O-H_3_PO_4_ are 73.2±1.2% s.e.m. at 1 A g^-1^, 85.1±2.3% s.e.m. at 2 A g^-1^, 95.8±3.4% s.e.m. at 5 A g^-1^, 98.8±2.6% s.e.m. at 10 A g^-1^, 99.1±3.4% s.e.m. at 15 A g^-1^, 96.7±15.1% s.e.m. at 20 A g^-1^, where n=5.


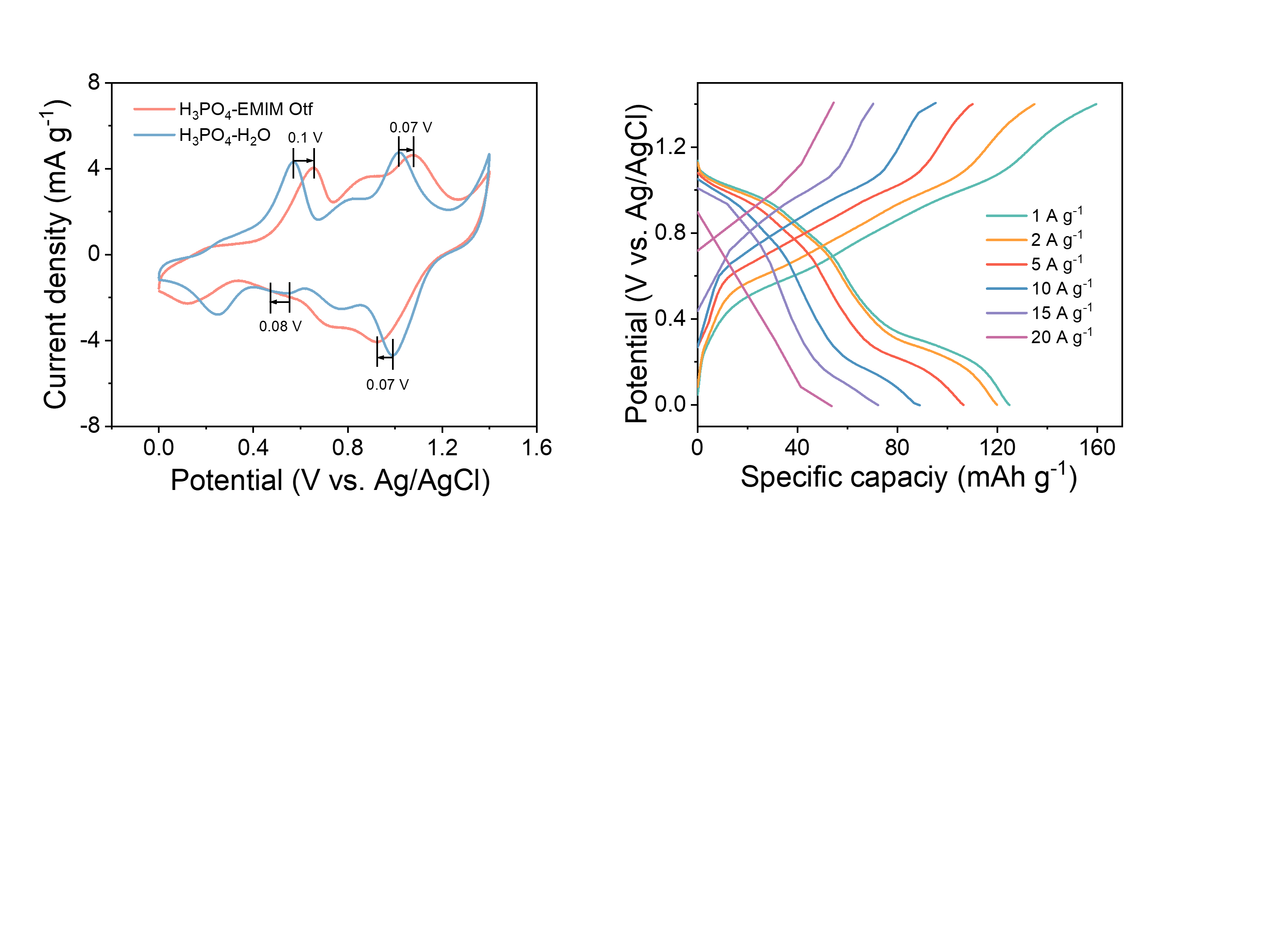


**Figure S12.** GCD curves of H-VHCF in H_2_O-H_3_PO_4_ at different current densities.


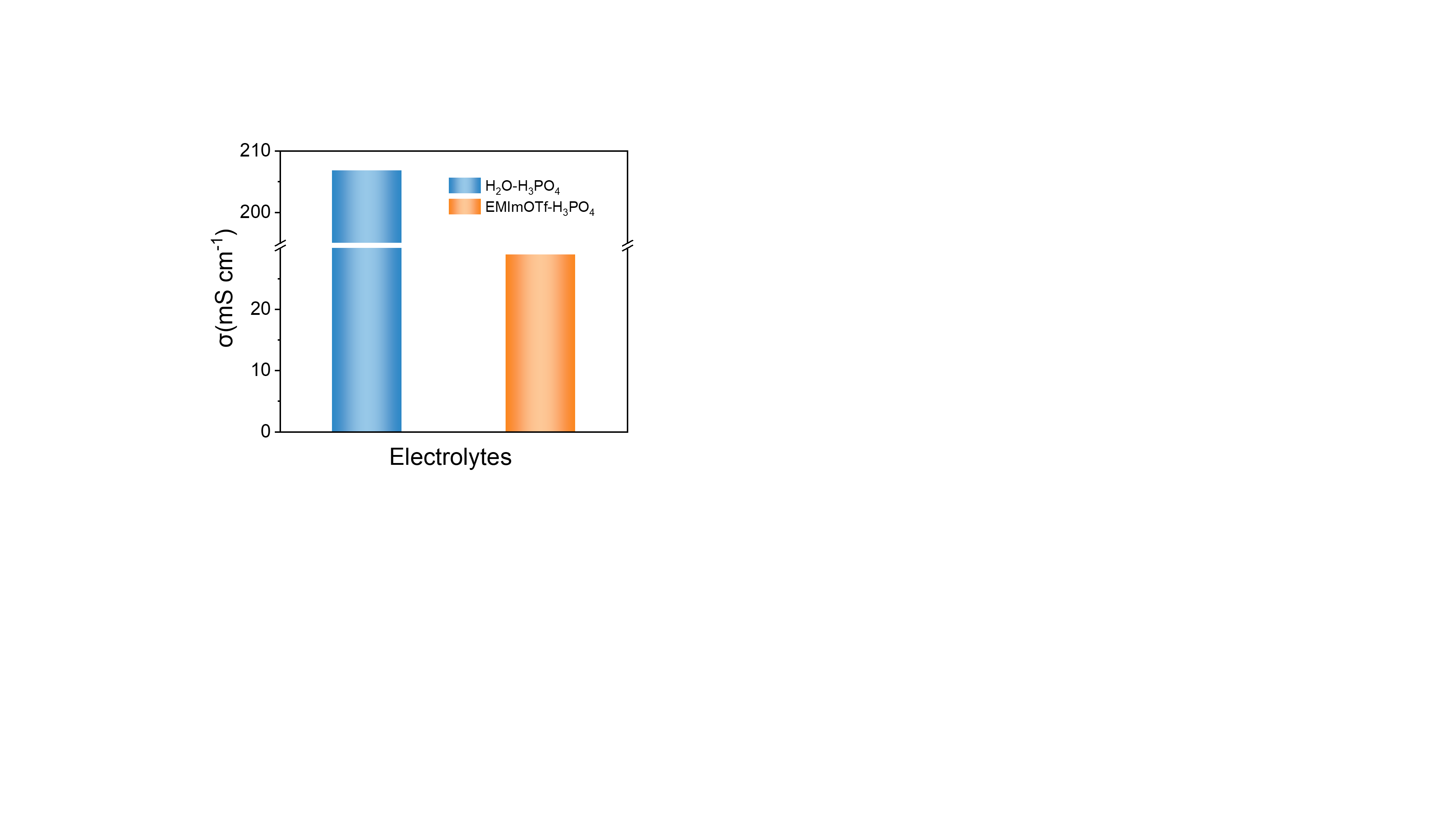


**Figure S13.** Proton conductivities of H_2_O-H_3_PO_4_ and EMImOtf-H_3_PO_4_.


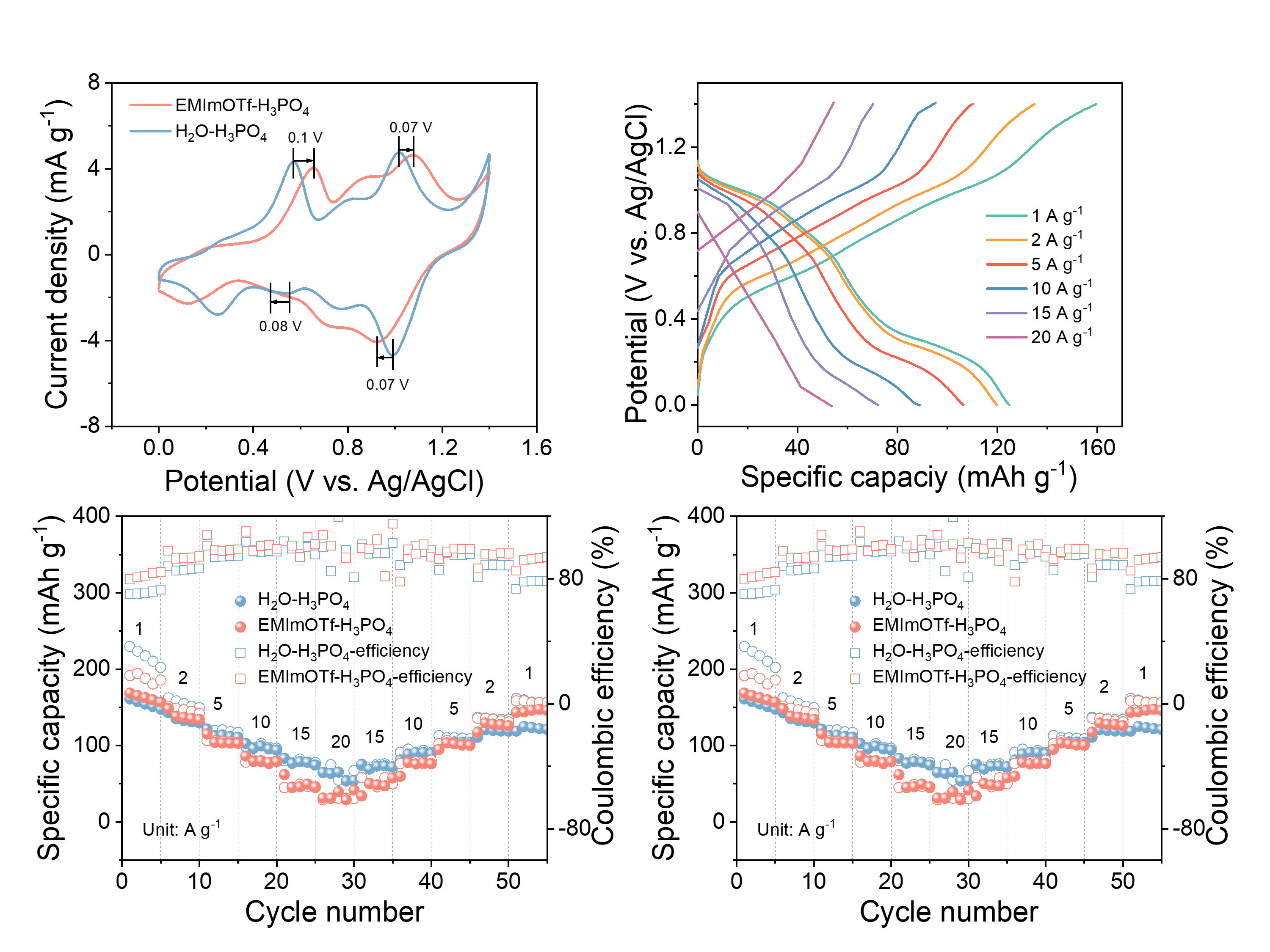


**Figure S14.** Rate performance of H-VHCF in EMImOTf-H_3_PO_4_ and H_2_O-H_3_PO_4_.


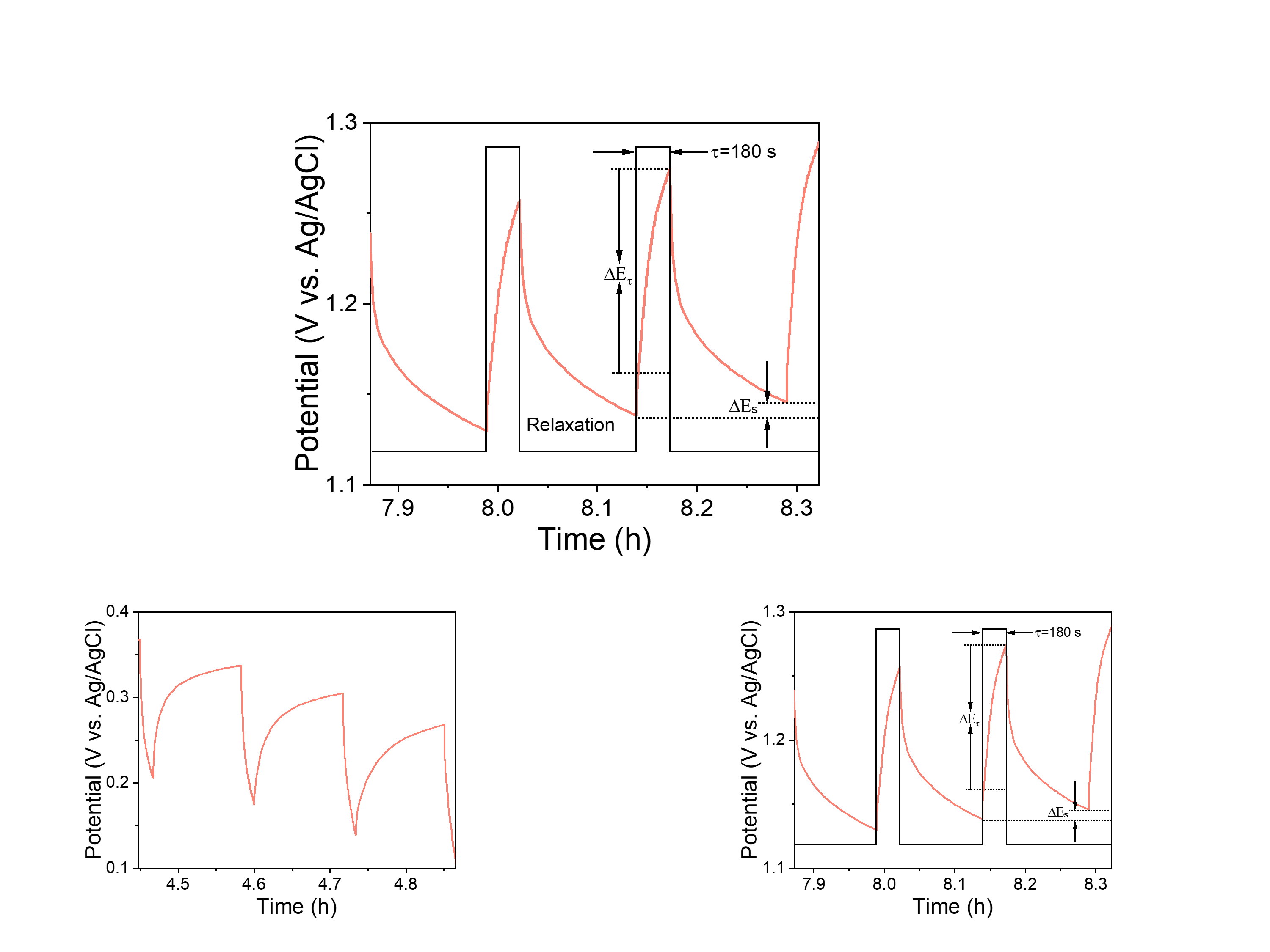


**Figure S15.** Close-pus of GITT curves for H-VHCF in the EMImOTf-H_3_PO_4_.


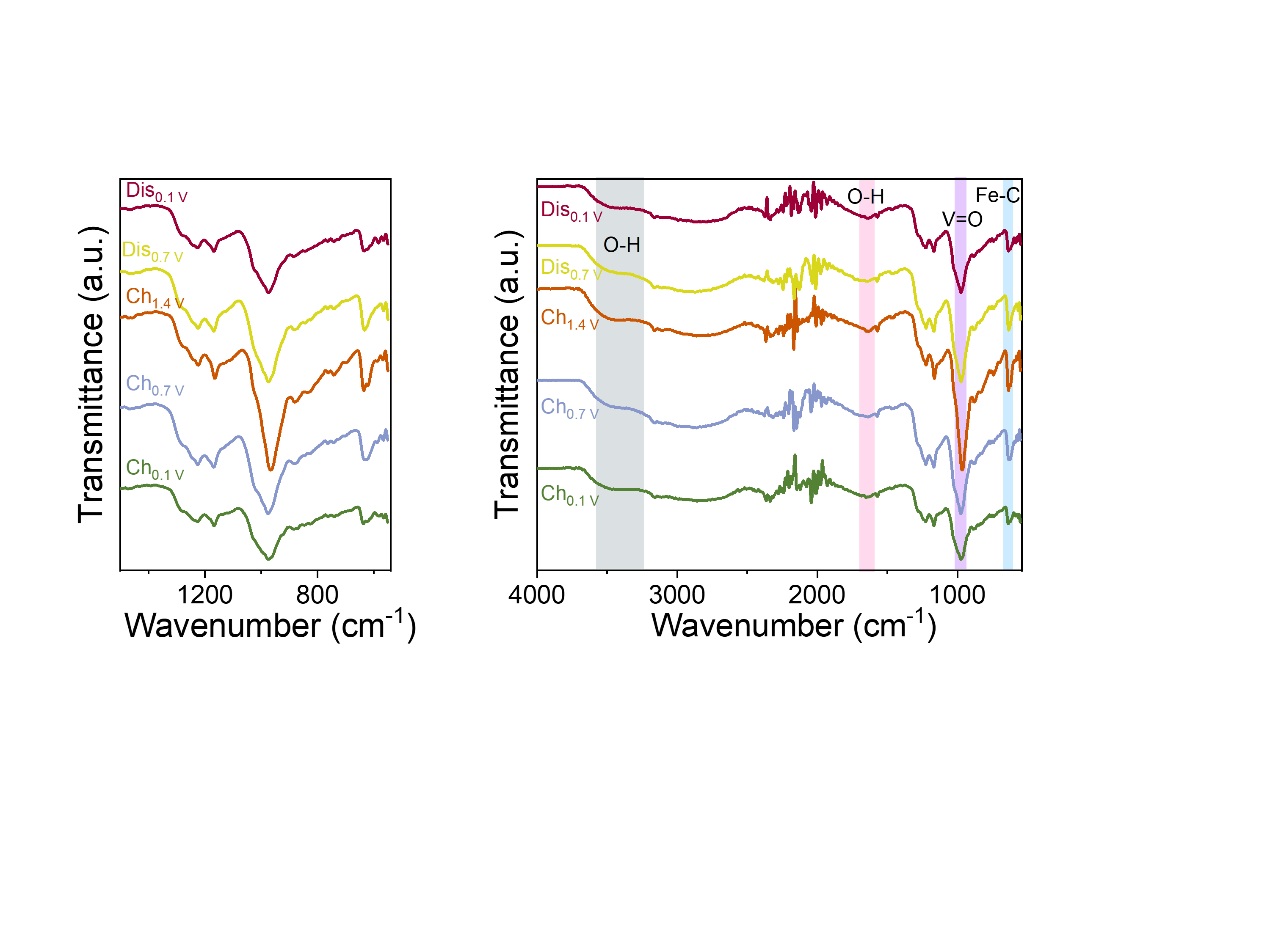


**Figure S16.** *In-situ* FT-IR spectra of H-VHCF in EMImOTf-H_3_PO_4_ in different charged and discharged states.


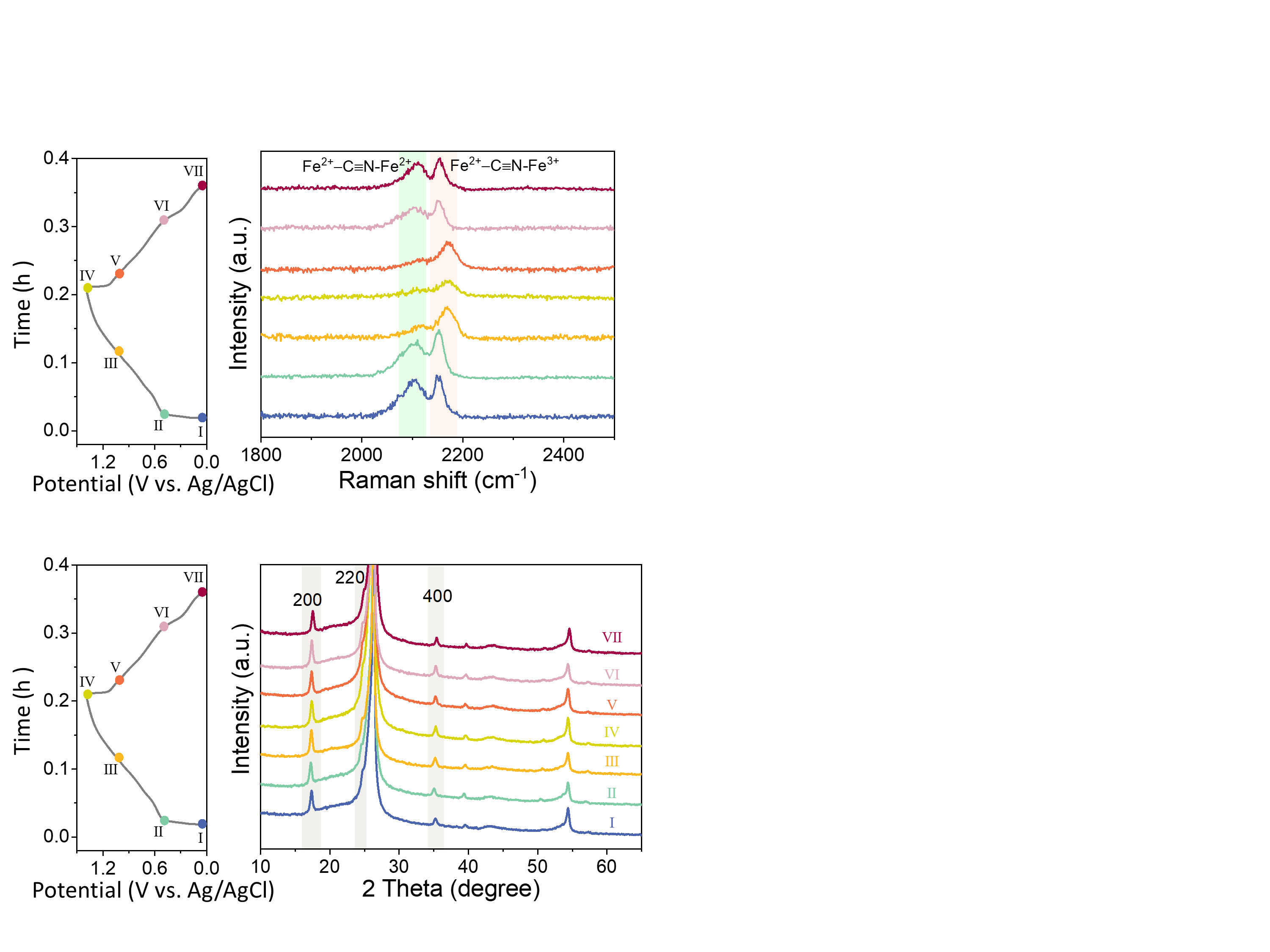


**Figure S17.** Time-potential curve and *ex-situ* Raman spectra of H-VHCF cathode in EMImOTf-H_3_PO_4_ electrolyte in different charged and discharged states.


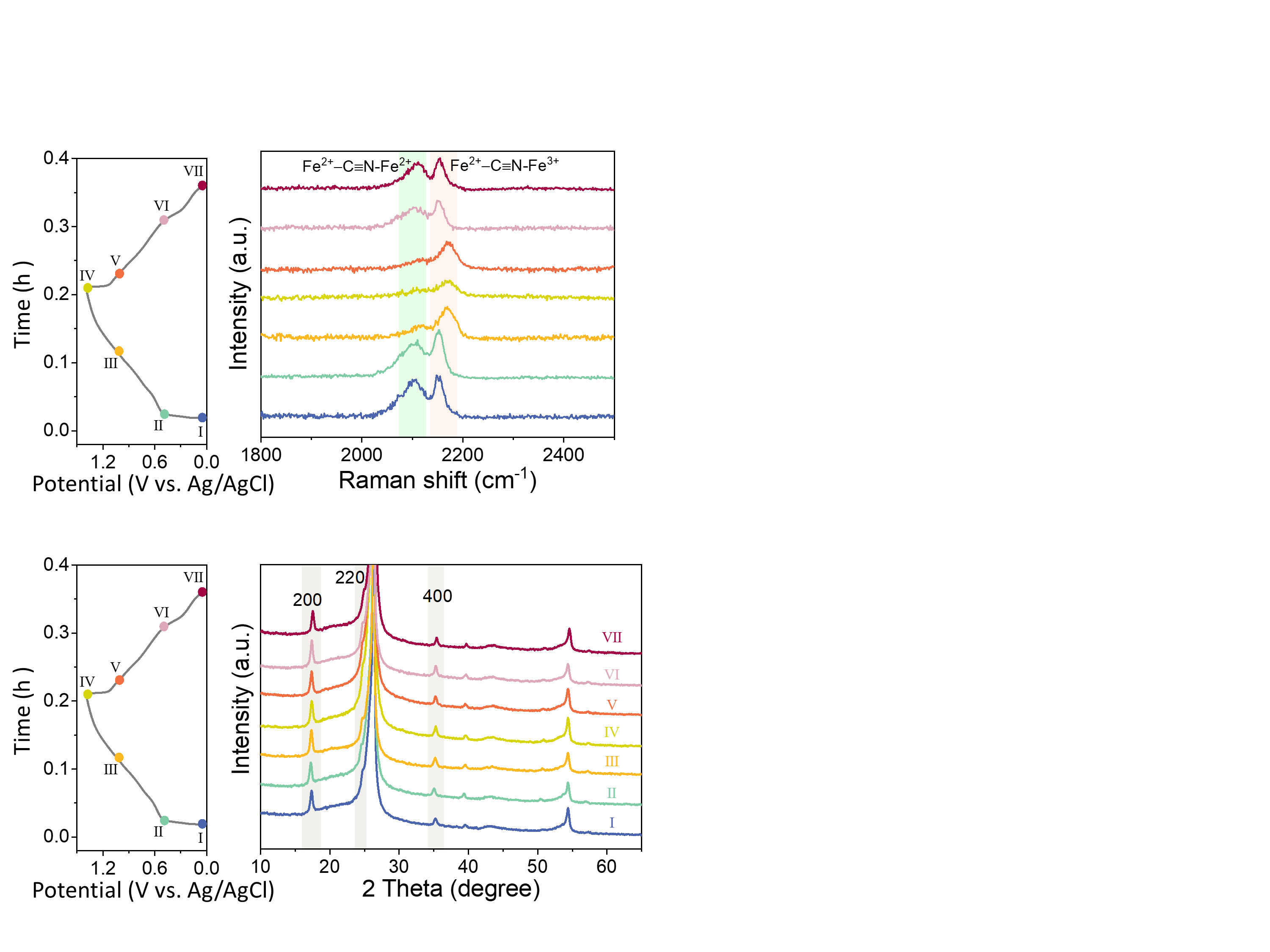


**Figure S18.** Time-potential curve and *ex-situ* XRD patterns of H-VHCF in EMImOTf-H_3_PO_4_ electrolyte in different charged and discharged states.


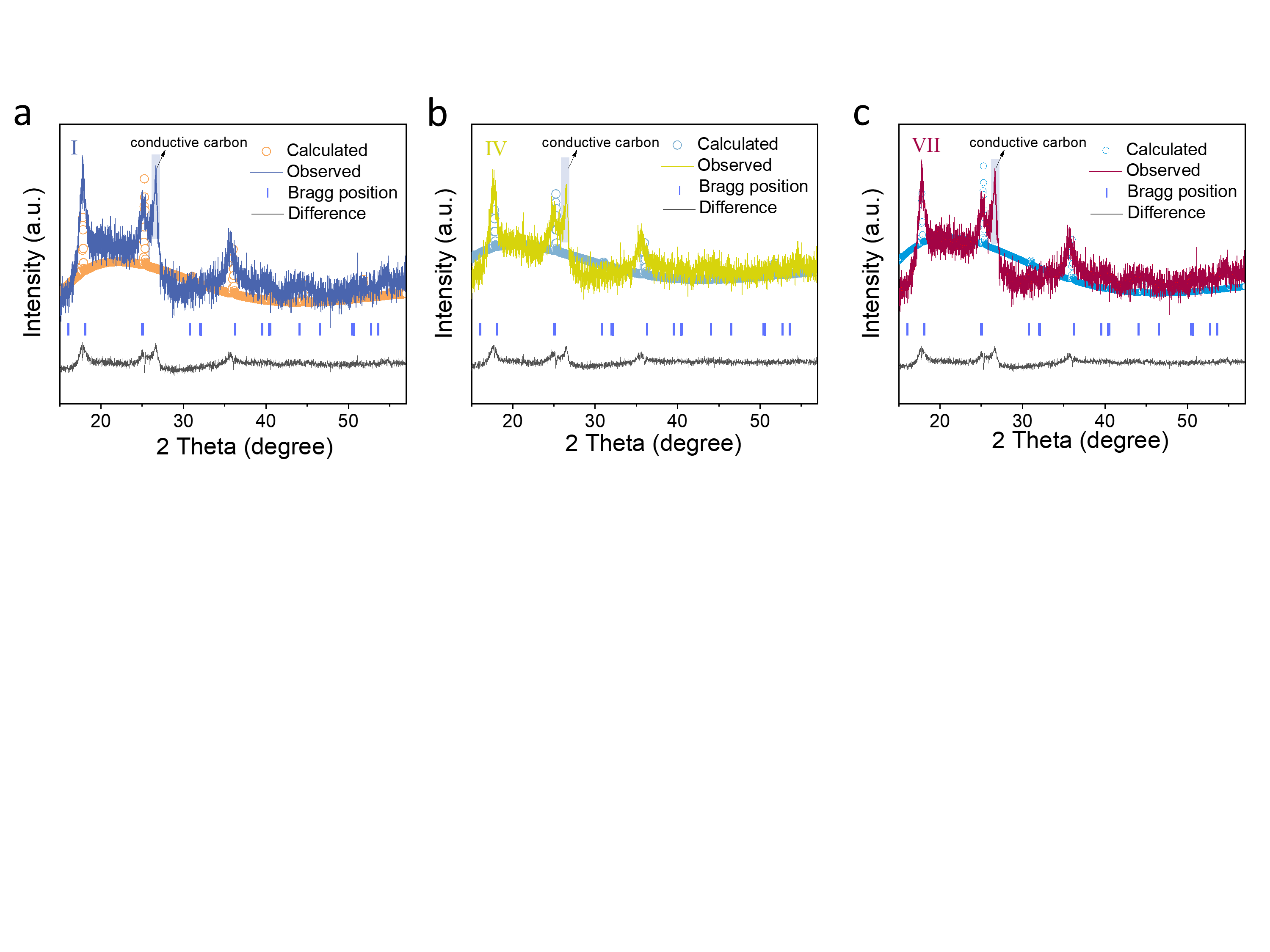


**Figure S19.** XRD Rietveld refinement profile of H-VHCF cathode in EMImOTf-H_3_PO_4_ electrolyte in the states of (a) Ⅰ, (b) IV, and (c) VII corresponding to the time-potential curves in Figure 3c.


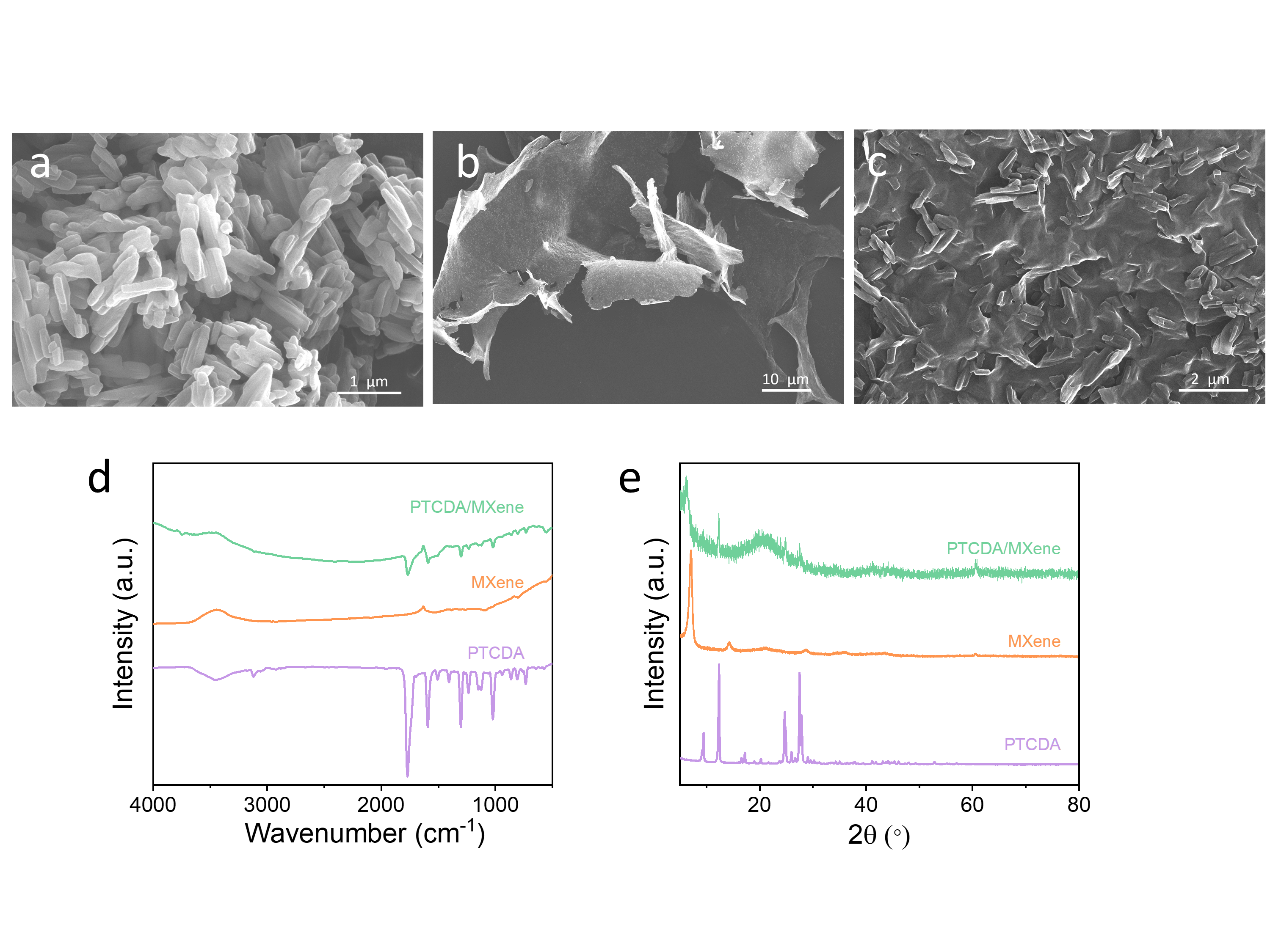


**Figure S20.** Physical characterization of PTCDA/MXene. SEM images of (a) PTCDA, (b) MXene, and (c) PTCDA/MXene. (d) FTIR spectra and (e) XRD curves of PTCDA, MXene and PTCDA/MXene.

SEM images reveal that PTCDA exhibits a short rod-like structure (Figure S20a), while MXene appears as thin sheets (Figure S20b). In the PTCDA/MXene composites, PTCDA nanorods are loaded onto the MXene sheets (Figure S20c). The composite material improves structural stability, lowering the risk of damage during the charge-discharge process. FTIR spectra reveal interactions between the characteristic functional groups of PTCDA and MXene (Figure S20d), indicating the successful preparation of the composite material. XRD patterns show that the crystal structures of the components in the composite remain intact (Figure S20e) with no significant peak shifts, indicating good compatibility and stable composite structure.


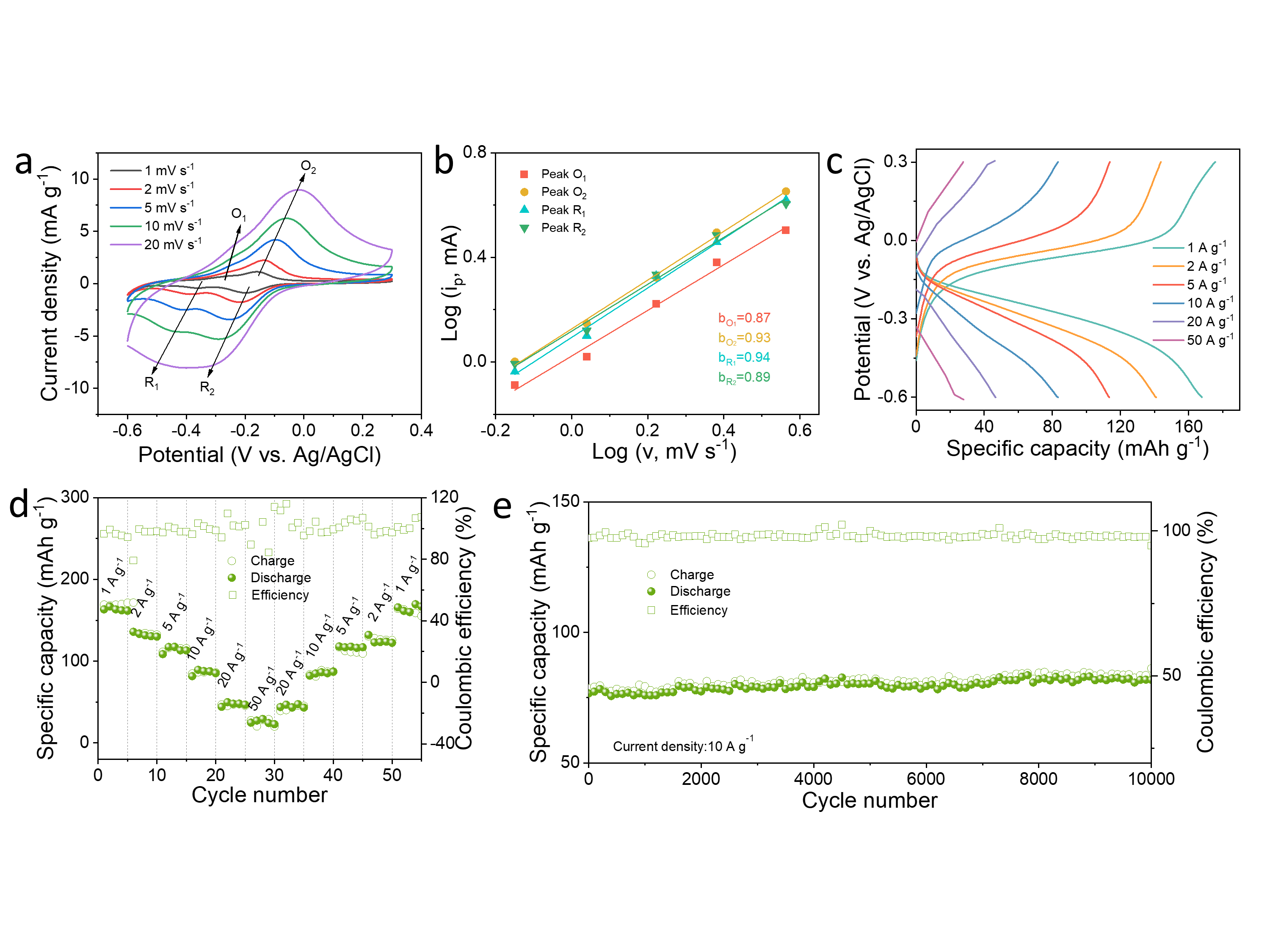


**Figure S21.** Electrochemical performance of PTCDA/MXene in EMImOTf-H_3_PO_4_ electrolyte. (a) CV curves at different scan rates. (b) Relationship between the peak current and scan rate. (c) GCD curves at different current densities. (c) Rate and (d) long-term cycling performance.

In CV curves, two pairs of redox peaks are observed, corresponding to the proton storage behavior of MXene (O_1_/R_1_) and PTCDA (O_2_/R_2_). The shape of the CV curves remains consistent even at a scan rate of 20 mV s^-1^ (Figure S21a), indicating excellent proton storage stability of the materials at high current. Figure S12b shows that the$b$ values of O_1_/R_1_ and O_2_/R_2_ are 0.87/0.94 and 0.93/0.89 respectively, indicating that capacitive storage predominates over proton storage in PTCDA/MXene (Figure S21b). This high capacitive contribution enhances the energy storage and response capability of the composite materials. The GCD curves at different current densities show that the specific discharge capacities of PTCDA/MXene are 167.8, 141.6, 113.1, 83.3, 46.6, and 27.8 mAh g^-1^ at 1, 2, 5, 10, 20, 50 A g^-1^, respectively (Figure S21c). After charging and discharging at high current densities and then returning to 1 A g^-1^, the capacity retains 96% of its initial value, indicating good resistance to high current (Figure S21d). After 10000 cycles, the capacity of PTCDA/MXene increased from 78.3 mAh g^-1^ to 81.9 mAh g^-1^ (Figure S21e), which is attributed to the gradual optimization of the material’s structure during cycling.


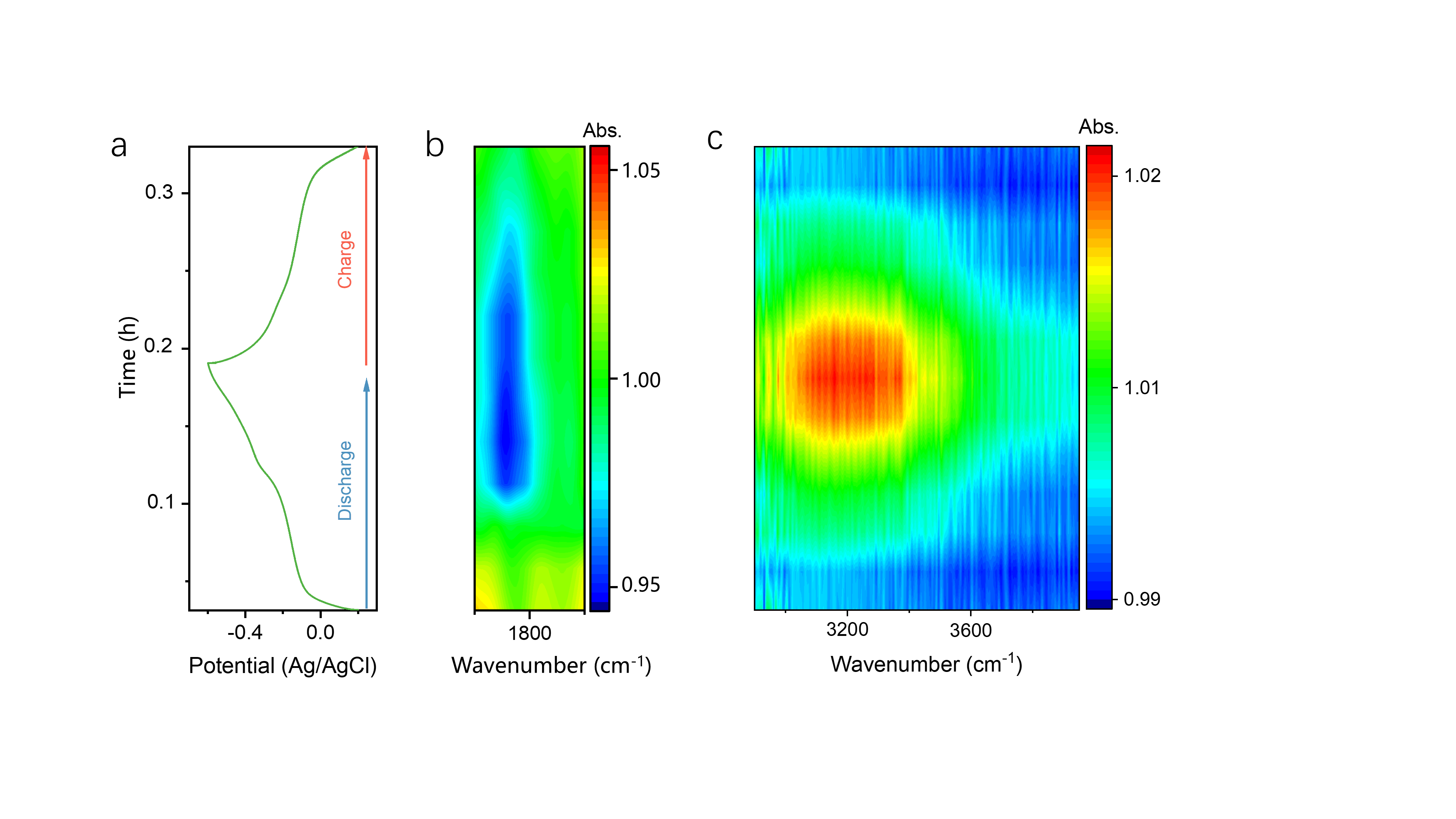


**Figure S22.** (a) GCD curves, (b) and (c) In-situ FTIR of of PTCDA/MXene with EMImOTf-H_3_PO_4_.

*In situ* FTIR spectra show that during the charging process, the C=O peak at 1768 cm^-1^ weakens, which is attributed to protonation, where C=O is converted to C-OH. During the discharging process, deprotonation causes the C=O peak to recover, indicating the reversible transition between C=O and C-OH (Figure S22a, Figure S22b). Additionally, the -OH peak at 3200 cm^-1^ intensifies during the discharging process and weakens during the charging process, demonstrating the good reversibility of the proton insertion and extraction process (Figure S22c).


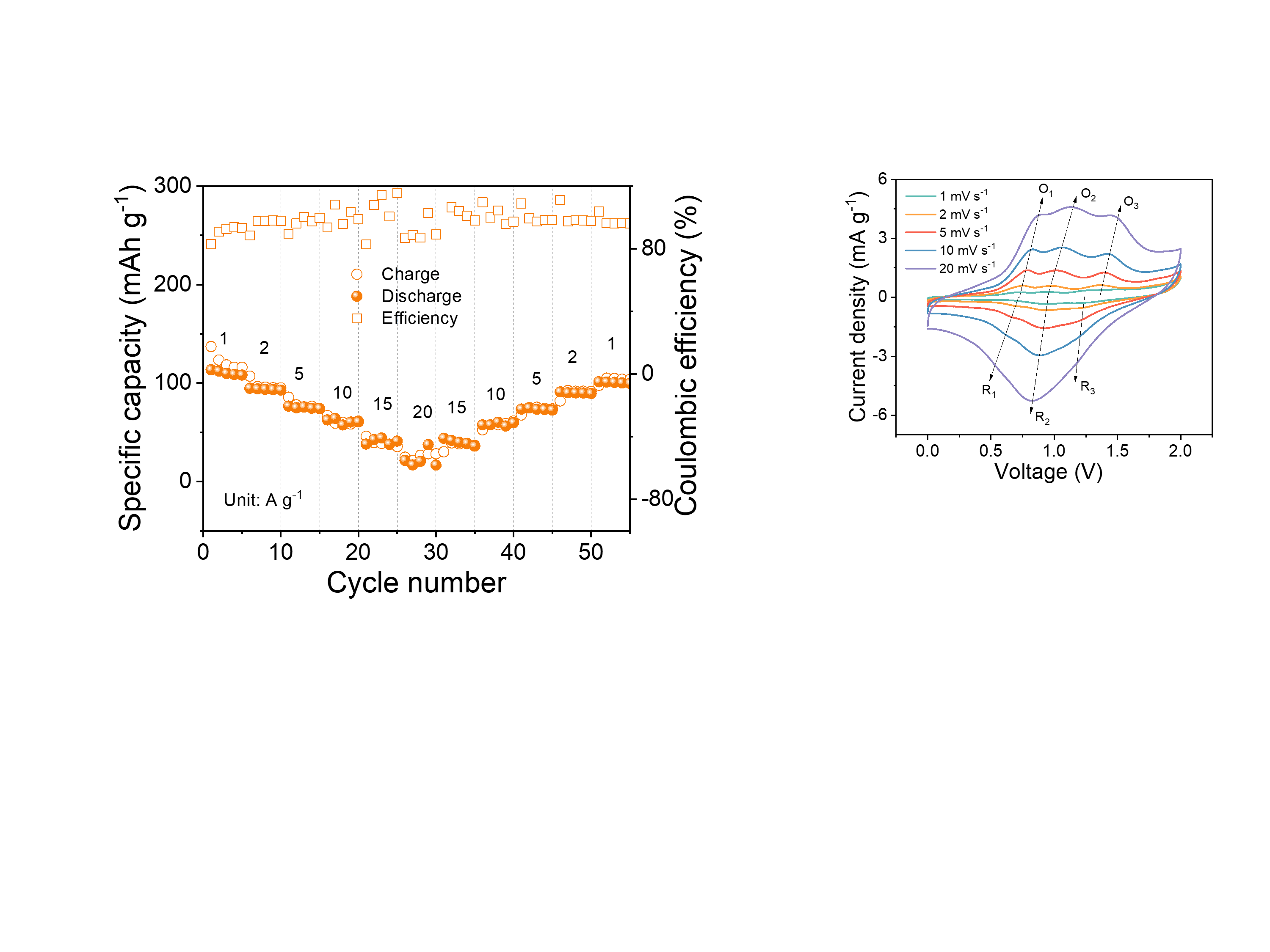


**Figure S23.** Rate performance of PTCDA/MXene//EMImOTf-H_3_PO_4_//H-VHCF full proton battery.


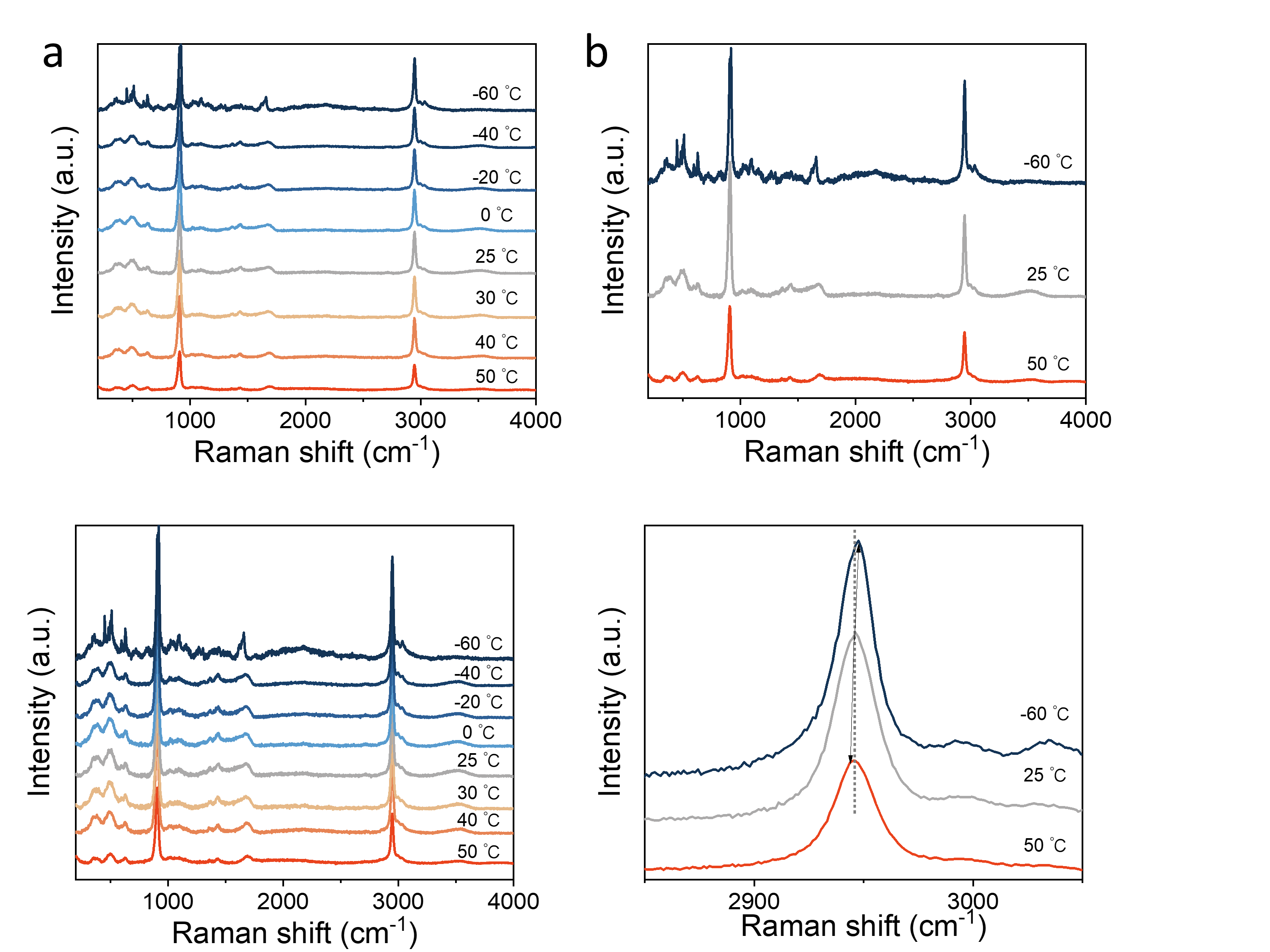


**Figure S24.** Raman spectra of EMImOTf-H_3_PO_4_ at a temperature range of (a) 50~-60 ℃ and (b) close-up views at 50, 25, and -60 ℃.


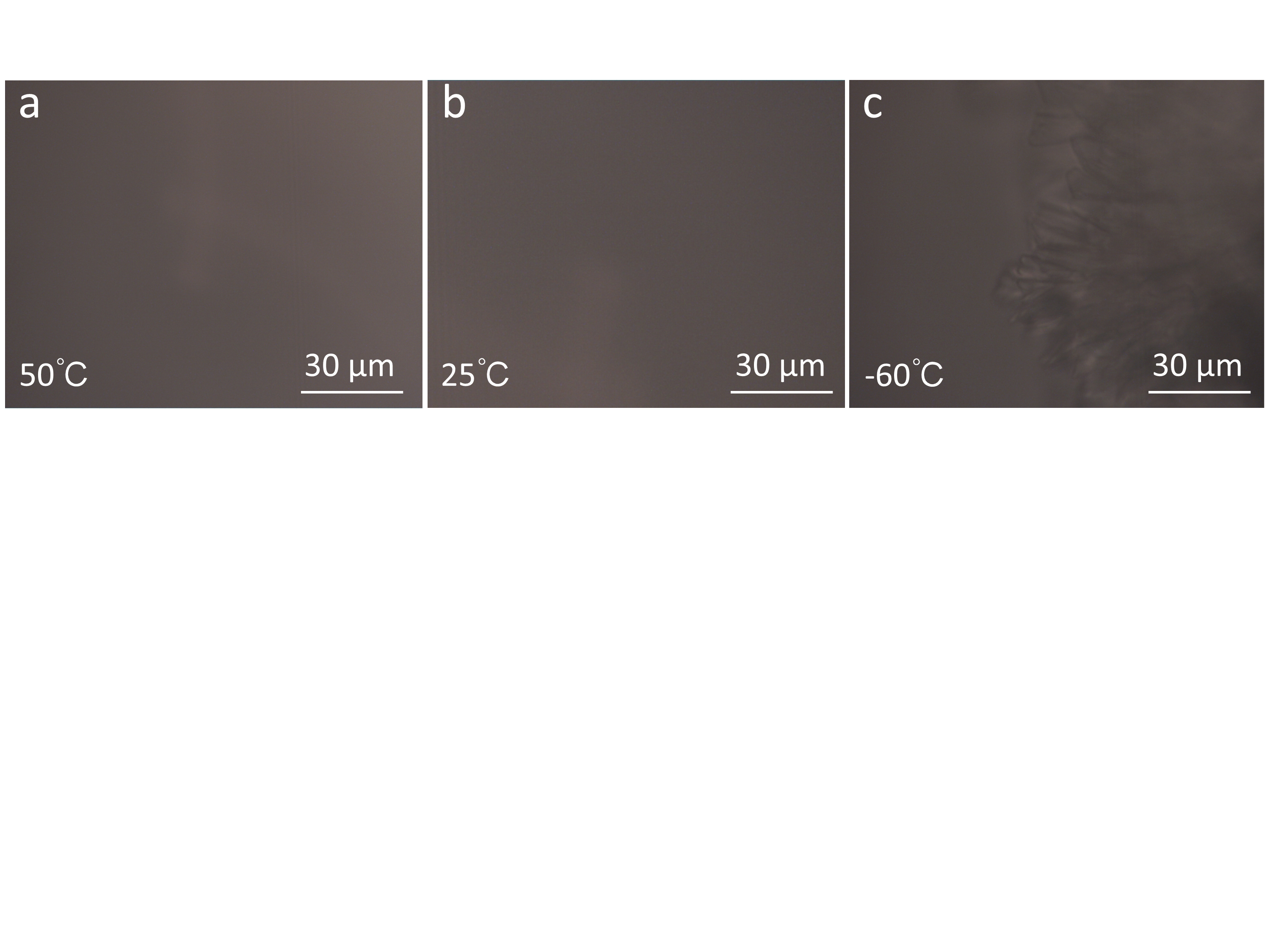


**Figure S25.** Digital photographs of EMImOTf-H_3_PO_4_ at (a) 50 ℃, (b) 25 ℃, and (c)

-60 ℃.


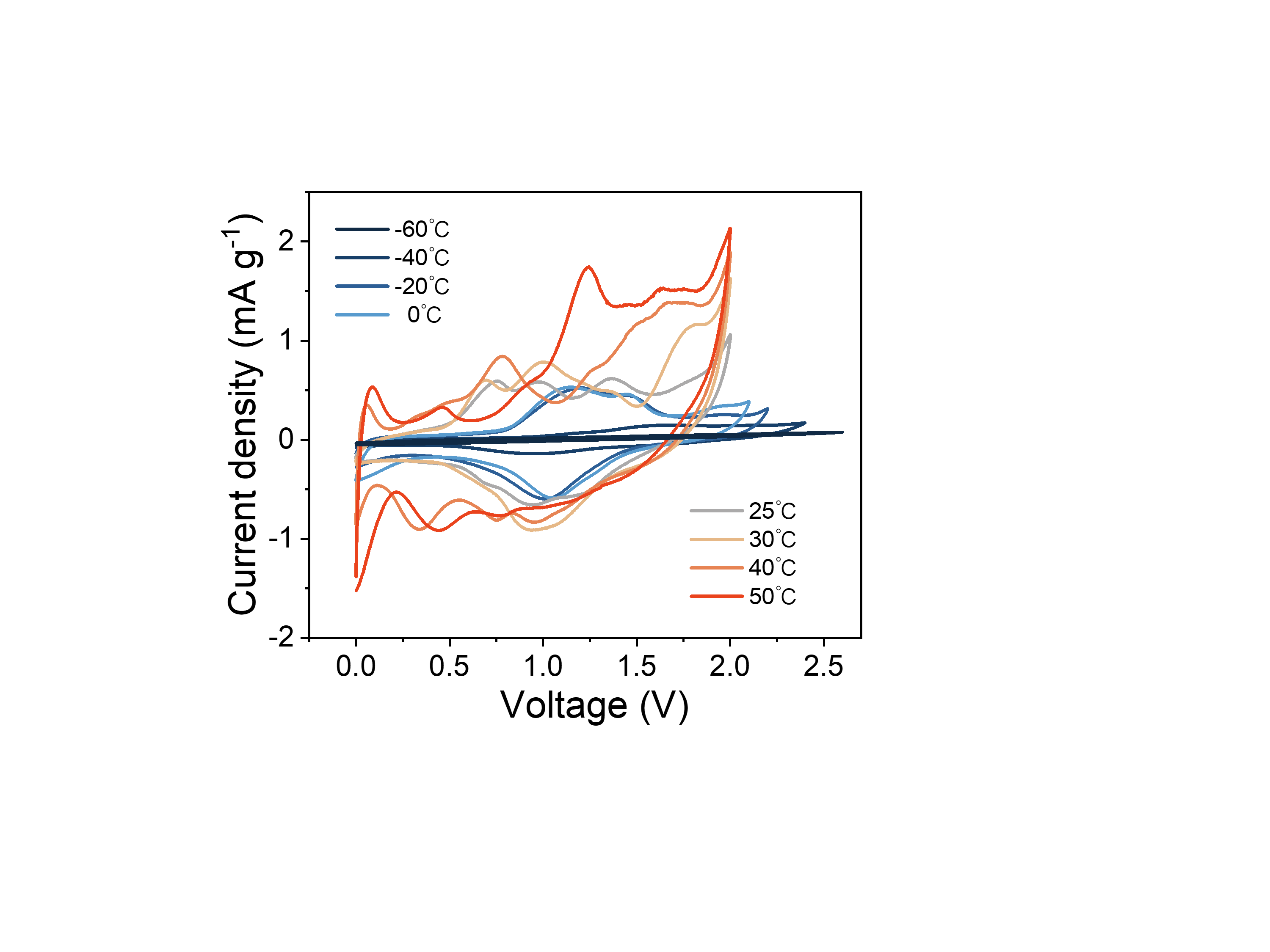


**Figure S26.** CV curves of PTCDA/MXene//EMImOTf-H_3_PO_4_//H-VHCF at different temperatures.


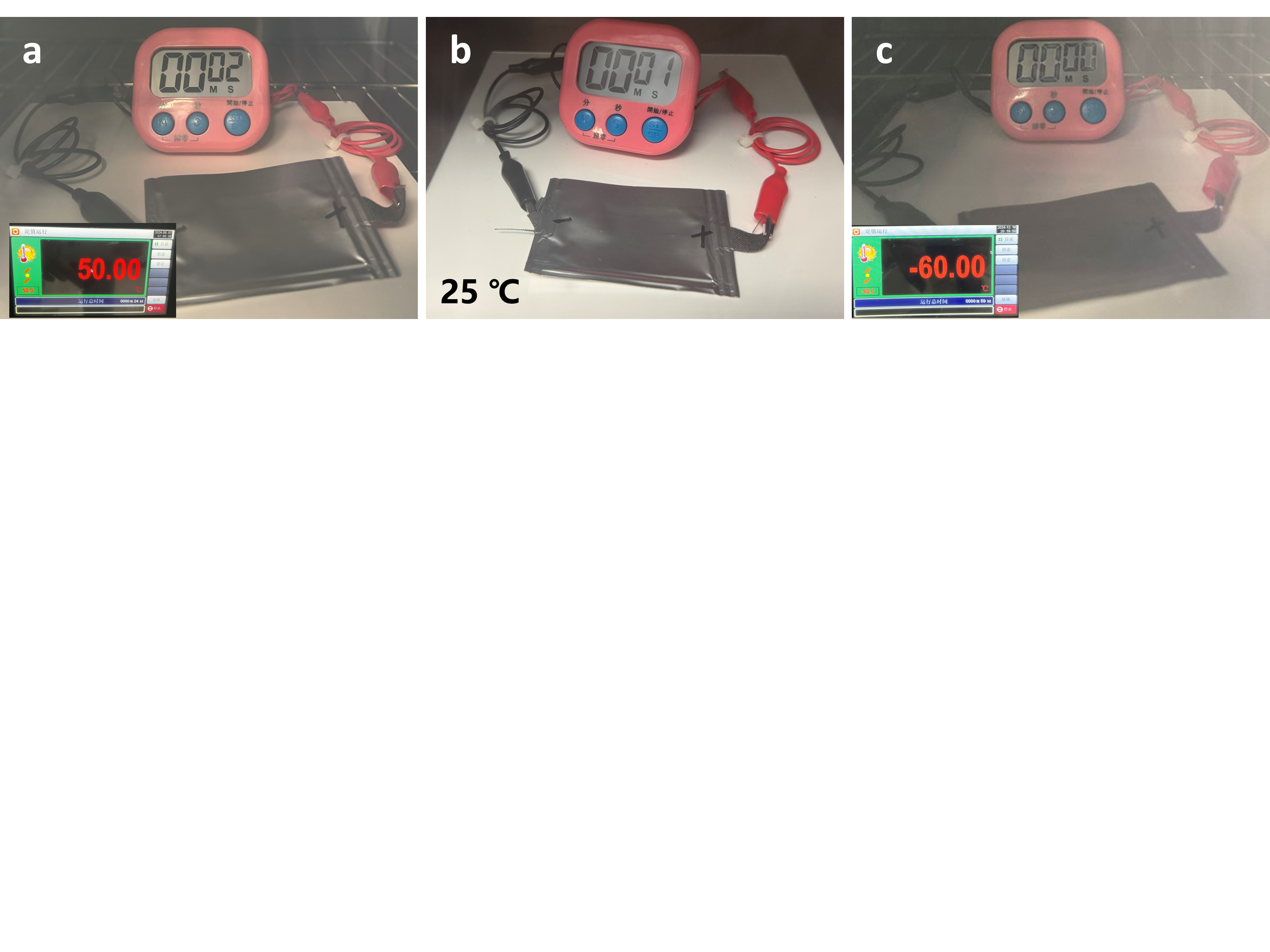


**Figure S27.** Digital images of a timer powered with a pouch cell at (a) 50 ℃, (b) 25 ℃, and (c) -60 ℃.


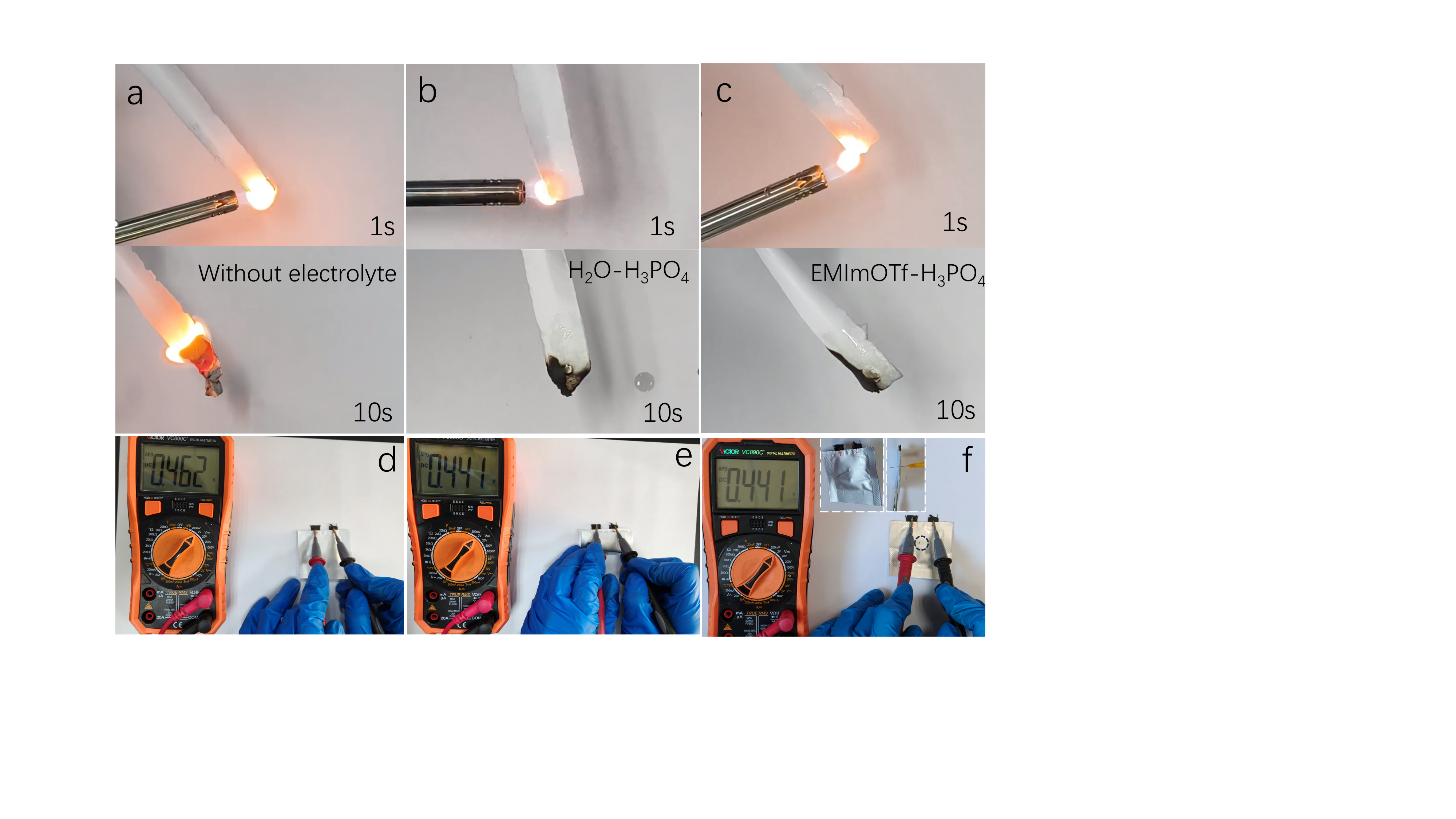


**Figure S28**. Safety tests. Digital images showing the combustion of (a) A4 paper without electrolyte, with (b) H_2_O-H_3_PO_4_, and (c) EMImOTf-H_3_PO_4_ electrolyte. Open-circuit voltage test of the pouch cell in the (d) initial state (e) after 180° folding, and (f) after puncture.

The combustion test results indicate that paper without any electrolyte easily catches fire, whereas paper impregnated with H_2_O-H_3_PO_4_ and EMImOTf-H_3_PO_4_ electrolytes cannot be ignited, demonstrating the excellent flame-retardant properties of the electrolytes (Figure S28a-c). Further open-circuit voltage tests of the pouch cell with EMImOTf-H_3_PO_4_ electrolyte reveals that neither folding the battery at 180° nor puncturing with a needle affect the open-circuit voltage (Figure S28d-f).

**Table S1.** Assignment of FTIR peaks of EMImOTf-H_3_PO_4_.

| **Wavenumber (cm^-1^)** | **Vibrational modes** | **Refs.** |
| --- | --- | --- |
| 3167 | Ip and op C_4,5_-H stretching | [1, 2] |
| 3116 | C_6_-H | [2, 3] |
| 1575 | Ring ip symmetric/asymmetric stretching, CH_2_(N) and CH_3_(N)CN stretching | [1, 2] |
| 1462 | Ring ip asymmetric stretching, CH_3(_N)CN stretching | [2, 3] |
| 1430 | Ring ip asymmetric stretching, CH_3(_N)CN stretching, CH_3_(N)HCH asymmetric bend | [1, 2] |
| 1238 | SO_3_ symmetric stretching | [2, 3] |
| 743 | CF_3_ bending group | [1, 3] |
| 640 | SO_3_ bending group | [3] |

Vibrational assignments: ip (in-plan), op (out-of-plan)

**Table S2.** Performance comparison of different full proton battery systems.

| **Systems** | **Electrolytes** | **Voltages (V)** | **Maximum energy densities (Wh kg^-1^)** | **Maximum power densities (W kg^-1^)** | **Cycle lifes** | **Refs.** |
| --- | --- | --- | --- | --- | --- | --- |
| RuO_2_//Hex-Aza-COF3 | 1 M H_2_SO_4_ | 0-1.4 | 23.3 | 661 | 3000 | [4] |
| PUQ/PTC | 0.5 M H_2_SO_4_ | 0-1.2 | 56.2 | 360 | 1000 | [5] |
| ACRG-800/ACRG-800-IND | 1 M H_2_SO_4_ | 0-1 | 13.2 | 1000 | 6000 | [6] |
| MoO_3_ NBs//NAC | 1 M H_2_SO_4_ | 0-1.6 | 46.2 | 16000 | 2300 | [7] |
| H-TBA/PS-MXene | 9.5 M H_3_PO_4_ | 0-1.5 | 26 | 16200 | 3000 | [8] |
| 2,6-DHAQ//2,6-DHN/rGO | 0.5 M H_2_SO_4_ | 0-1.2 | 70.8 | 850 | 1000 | [9] |
| 2,6-DHAQ//2,6-DHN | 0.5 M H_2_SO_4_ | 0-1.2 | 50.4 | 850 | 1000 | [9] |
| PTCDA/MXene //EMImOTf-H_3_PO_4_// H-VHC | 8.5 M EMImOTf-H_3_PO_4_ | 0-2 | 87.5 | 30632 | 30000 | This work |

**Table S3.** Temperature range comparison of different full proton battery systems.

| **System** | **Electrolytes** | **Voltage (V)** | **Temperature range (℃)** | **Cycle life** | **Ref.** |
| --- | --- | --- | --- | --- | --- |
| pEP(QH_2_)E// pEP(NQ)E | 0.5 M H_2_SO_4_ | 0.1-0.6 | -24-25 | 500 | [10] |
| P-AS//AN-PA | 2 M HCl | 0-1 | -25-25 | 1000 | [11] |
| PANI//CF | 3.5 M Mn(ClO_4_)_2_ | 0.2-1.5 | -70-25 | 100 | [12] |
| WO_3_🞄0.6 H_2_O//Vfe-PBA | 4.5 M H_2_SO_4_+3 M glycerol | 0-1.6 | -50-25 | 100 | [13] |
| MoO_3_ NBs//NAC | 1 M H_2_SO_4_ | 0-1.6 | -25-65 | - | [7] |
| H-VHCF//  MoO_3_/MXene | 8.5 M H_3_PO_4_ | 0-1.5 | -40-25 | 1000 | [14] |
| VHCF//h-WO_3_ | 9.5 M H_3_PO_4_ | 0-2 | -60-25 | 1000 | [15] |
| MoO_3_//VHCF | LIE (H_3_PO_4_+TMP) | 0-1.6 | -20-25 | 3500 | [16] |
| H-TBA//PS-MXene | 9.5 M H_3_PO_4_ | 0-1.5 | -60-20 | - | [8] |
| PTCDA/MXene //EMImOTf-H_3_PO_4_// H-VHC | 8.5 M EMImOTf-H_3_PO_4_ | 0-2.4 | -60-50 | 7000 | This work |

**References**

[1] R. Rathika and S. A. Suthanthiraraj*.* *J. Mater. Sci-Mater. El.*, **2018**, 29, 19632-19643.

[2] Pandey, G.P., S.A. Hashmi, and R.C*.* *J. Phys. D: Appl. Phys.*, **2008**, 41, 055409.

[3] K. Noack, P. S. Schulz, N. Paape, J. Kiefer, P. Wasserscheid, A. Leipertz, *Phys. Chem. Chem. Phys.*, **2010**, 12, 14153-61.

[4] S. Kandambeth, J. Jia, H. Wu, V. S. Kale, P. T. Parvatar, J. C. jozwiak, S. Zhou, X. Xu, Z. O. Ameur, E. A. Hamad, O. Shekhah, H. N. Alshareef, M, *Adv. Energy Mater.*, **2020**, 10, 2001673

[5] M. Zhu, L. Zhao, Q. Ran, Y. Zhang, R. Peng, C. Lu, X. Jia, D. Chao, C. Wang, *Adv. Sci. (Weinh)*, **2022**, 9, 2103896.

[6] S. Brahma, and K. Ramanujam, *Ionics*, **2022**, 28, 1427-1440.

[7] Y. Wu, W. Liu, Z. Zhang, Y. Zheng, X. Fu, J. Lu, S. Cheng, J. Su, Y. Gao, *Energy Stor. Mater.*, **2023**, 61,102849.

[8] J. Zhu, X. Li, B. Hu, S. Ge, J. Xu, *Batteries*, **2024**, 10, 207.

[9] G. Zhao, X. Yan, Y. Dai, J. Xiong, Q. Zhao, X. Wang, H. Yu, J, Gao, N. Zhang, M. Hu, J. Yang, *Small*, **2024**, 20, 2306071.

[10] C. Strietzel, M. Sterby, H. Huang, M. Stromme, R. Emanuelssion, M. Sjodin, An *Angew. Chem. Int. Ed. Engl.*, **2020**, 59, 9631-9638.

[11] K.C.S. Lakshmi, B. Vedhanarayanan, H. Cheng, X. Ji, H. Shen, T. Lin, *J. Colloid Interface Sci.*, **2022**, 619, 123-131.

[12] T. Sun, Q. Hian, H. Du, S. Zheng, D. Han, Z. Tao, *J. Mater. Chem. A*, **2022**, 10, 17288-17296.

[13] Z. Su, J. Chen, J. Stansby, C. Jia, T. Zhao, J. Tang, Y. Fang, A. Rawal, J. Ho, C. Zhao, *Small*, **2022**, 15, 2201449.

[14] X. Dong, Z. Li, D. Luo, K. Huang, H. Dou, X. Zhang, *Adv. Funct. Mater.*, **2023**, 2210473

[15] Z. Cui, Di Wang, T. Zhu, T. Yao, L. Shen, *Chem. Eng. J.*, **2024**, 495, 153347

[16] P. Liang, S. Di, Y. Zhu, Z. Li, S. Wang, L. Li, *Angew. Chem. Int. Ed. Engl.*, **2024**, e202409871.
